# Nuclear calcium signatures are associated with root development

**DOI:** 10.1038/s41467-019-12845-8

**Published:** 2019-10-25

**Authors:** Nuno Leitão, Pierre Dangeville, Ross Carter, Myriam Charpentier

**Affiliations:** 1 Department of Cell and Developmental Biology, John Innes Centre, Colney Lane, Norwich, NR4 7UH UK; 2 The Sainsbury Laboratory, University of Cambridge, Cambridge, CB2 1LR UK; 3 Present Address: Synthace Ltd, The Westworks, London, W12 7FQ UK

**Keywords:** Plant sciences, Plant signalling

## Abstract

In plants, nuclear Ca^2+^ releases are essential to the establishment of nitrogen-fixing and phosphate-delivering arbuscular mycorrhizal endosymbioses. In the legume *Medicago truncatula*, these nuclear Ca^2+^ signals are generated by a complex of nuclear membrane-localised ion channels including the DOES NOT MAKE INFECTIONS 1 (DMI1) and the cyclic nucleotide-gated channels (CNGC) 15s. *DMI1* and *CNCG15s* are conserved among land plants, suggesting roles for nuclear Ca^2+^ signalling that extend beyond symbioses. Here we show that nuclear Ca^2+^ signalling initiates in the nucleus of Arabidopsis root cells and that these signals are correlated with primary root development, including meristem development and auxin homeostasis. In addition, we demonstrate that altering genetically *AtDMI1* is sufficient to modulate the nuclear Ca^2+^ signatures, and primary root development. This finding supports the postulate that stimulus-specific information can be encoded in the frequency and duration of a Ca^2+^ signal and thereby regulate cellular function.

## Abstract (web summary)

Nuclear Ca2+ signals generated by DMI1 and CNGC15 ion channels in plant roots are needed to establish symbiotic relationships with soil microbes. Here Leitão et al. show that DMI1- dependent nuclear Ca2+ spiking also occurs in Arabidopsis root meristems and are linked to root development

## Introduction

Calcium (Ca^2+^) is a universal regulatory element that intimately couples primary biotic and abiotic signals to many cellular processes, allowing plants and animals to develop and adapt to environmental stimuli^1,2^. Each Ca^2+^ response can be specified by the spatial release of Ca^2+^ in specific cell types and cellular compartments, which enables the compartmentalisation of the signal transduced and/or the selective activation of temporally and spatially regulated Ca^2+^-binding proteins. Within the same cellular compartment, it is postulated that the Ca^2+^ signature, defined by its amplitude, frequency, and duration, specifies the activation of downstream components^2^. Although this postulate is well accepted, the genetic demonstration that modulating a Ca^2+^ signature can change a specific developmental or physiological process is still missing.

Ca^2+^ signals generated autonomously by the nucleus govern cellular functions in animals such as cell proliferation^3^, cardiomyocyte hypertrophy^4^, neuronal gene expression, and neuroprotection^5^. In plants, nuclear Ca^2+^ signals are essential to establish nitrogen-fixing and phosphate-delivering arbuscular mycorrhizal (AM) endosymbioses^6^. The nuclear Ca^2+^ release is mediated by a complex of nuclear membrane-localised ion channels including DOES NOT MAKE INFECTIONS 1 (DMI1) and the cyclic nucleotide-gated channels (CNGC) 15a, b, c^7^. The conservation of *DMI1* and *CNCG15s* among all land plants^7,8^, including non-symbiotic species such as *Arabidopsis thaliana*, suggests roles for nuclear Ca^2+^ signalling that extend beyond symbioses.

Here we demonstrate, using a dual-localised fluorescent Ca^2+^ sensor, discriminating Ca^2+^ release within the nucleus versus cytoplasmic Ca^2+^ signals diffusing into the nucleus^9^ and that Ca^2+^ release occurs in the nucleus of root meristematic cells. We further demonstrate that the nuclear Ca^2+^ signal can be modulated genetically by removing or overexpressing the cation channel DMI1, leading to the production of different nuclear Ca^2+^ signatures and differences in primary root growth. In addition, we provide evidence that nuclear Ca^2+^ signals contribute to and can be modulated by root auxin homoeostasis. The findings in this study reveal that genetically modulating the activity of ion channels to produce diverse Ca^2+^ signatures is sufficient to control developmental processes and further highlight the novel function of DMI1 in modulating nuclear Ca^2+^ signals required for primary root growth.

## Results

### AtDMI1 and AtCNGC15 localise in root cell nuclear envelope

Phylogenetic analyses of the *Medicago truncatula DMI1* and *CNGC15a,b,c* identified Arabidopsis *DMI1* (At5g49960) and *CNGC15* (At2g28260) as orthologues of *MtDMI1* and *MtCNGC15b,c*, respectively^7,8^. AtDMI1 was previously characterised as the functional analogue of MtDMI1 in root symbioses^10^. Similarly, expression of *AtCNGC15* in the *Mtcngc15b,c* double mutant restores the symbiotic defect (Supplementary Fig. 1), demonstrating that AtCNGC15 is the functional analogue of MtCNGC15b,c. To gain insight into the function of AtDMI1 and AtCNGC15, we explored the transcriptomic database of Arabidopsis^11–13^. Transcriptomic data suggest that *AtDMI1* and *AtCNGC15* are expressed in diverse Arabidopsis tissues. We purified mRNA from roots, leaves, stems, and siliques of Arabidopsis and confirmed by reverse transcription quantitative polymerase chain reaction (RT-qPCR) that both *AtDMI1* and *AtCNGC15* are expressed in all tissues analysed including roots (Supplementary Fig. 2). To further characterise the expression pattern of *AtCNGC15* and *AtDMI1*, we generated transgenic lines expressing *β-GLUCURONIDASE* (*GUS*) and green fluorescent protein (*GFP*) under the control of the promoters of *AtCNGC15* and *AtDMI1*, respectively, and showed that these genes are expressed in the primary root apical meristem (Supplementary Figs. 3 and 4a). It is also noteworthy that *AtCNGC15* is expressed constitutively in cotyledons and specifically in guard cells in *A. thaliana* leaves (Supplementary Fig. 3e–g). Furthermore, expression of *AtCNGC15* and *AtDMI1* fused to *GFP* demonstrate that both AtCNGC15 and AtDMI1 localise in the nuclear envelope of root meristematic cells (Supplementary Fig. 4).

### AtDMI1 is required for primary root growth

**Fig. 1:**
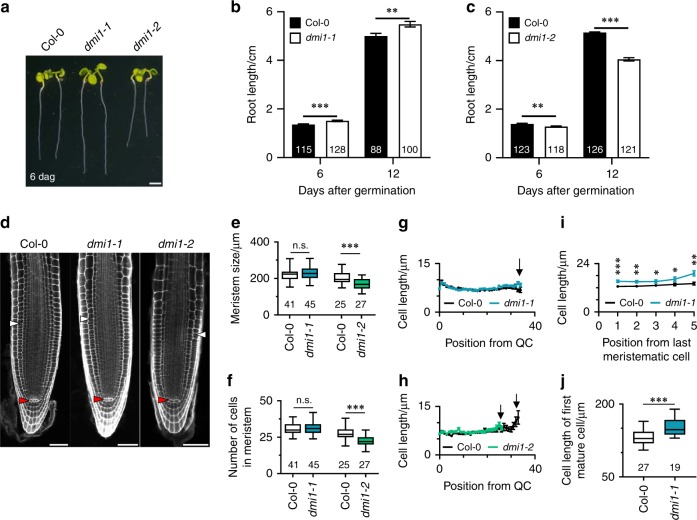
*dmi1* mutants are impaired in primary root development. **a** Representative image of Col-0, *dmi1-1*, and *dmi1-2* seedlings 6 days after germination (dag) (scale bar represents 0.2 cm). **b**, **c** Primary root length of wild type (Col-0), *dmi1-1* (**b**) and *dmi1-2* (**c**) 6 and 12 dag. **d** Cellular organisation of the root meristem visualised by confocal microscopy after staining with propidium iodide of wild type (Col-0), *dmi1-1*, and *dmi1-2* at 6 dag. White and red triangles mark the first elongated cortex cell and the quiescent centre (QC), respectively. Scale bars represent 50 µm. **e**–**h** Root meristem length (**e**), root meristem cell number (**f**), and cell length over cell position from the QC to the last meristematic cortex cell (**g**, **h**) of wild type (Col-0), *dmi1-1*, and *dmi1-2*. Black arrows in **g**, **h** mark the last meristematic cell. **i** Cell length over cell position from the first rapidly elongated cortex cell of Col-0 and *dmi1-1*. (*n* ≥ 41 in each population for each cell position)*.* **j** Cell length of the first mature cortex cell of Col-0 and *dmi1-1*. Values in bar and *xy* charts are means ± s.e.m. Box and whisker plots show 25% and 75% percentiles, median, minimum, and maximum. Numbers in bars and under boxes denote sample size (*n*). n.s. not significant, *p < 0.05, **p < 0.01, ***p < 0.001 (two-tailed *t* test with a prior *F*-test for homoscedasticity). **b**, **c**, **e**–**i** The data represent three biological replicates. **j** The data represent two biological replicates combined

To investigate the function of the nuclear-localised ion channel, AtDMI1, we identified mutant alleles in which the expression of *AtDMI1* is impaired (*dmi1-1*) or increased (*dmi1-2*) (Supplementary Fig. 5a–c). To assess how the mutant alleles were affected by these mutations, we monitored root growth over 12 days and revealed that *dmi1-2* have shorter primary roots (Fig. 1a, c, d). Analyses of meristem cellular organisation indicated that the defect in the primary root length reflects a reduction in the number of meristematic cells resulting in the establishment of a shorter root meristem (Fig. 1e, f, h). To validate that AtDMI1 is responsible for the root length defect, we mimicked the *dmi1-2* phenotype by overexpressing the *AtDMI1* genomic sequence in the wild-type background (Supplementary Fig. 6). In addition, in correlation with the overexpression of *AtDMI1* leading to shorter roots, the loss of function *AtDMI1* mutant (*dmi1-1*) displayed longer roots, associated with longer elongating and mature cells (Fig. 1a, b, e–g, I, j). Expression of the coding sequence of *AtDMI1* restored the root length and the elongating cell size of *dmi1-1* plants to wild-type levels (Supplementary Fig. 7a, b). Together, these data indicate that AtDMI1 is required for root apical meristem development and thus for primary root growth.

### Nuclear Ca^2+^ signals are generated in root meristem cells

**Fig. 2:**
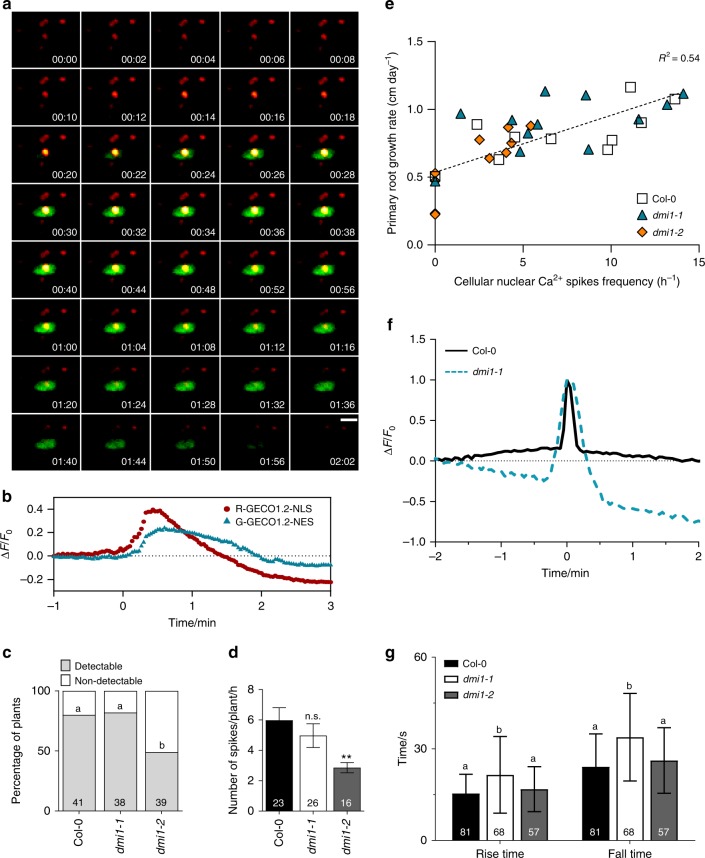
Nuclear Ca^2+^ signals occur during root growth and are dependent on DMI1 in *Arabidopsis thaliana*. **a** Representative growth-induced Ca^2+^ signal in a cell in the root tip of a 5-day-old wild-type seedling expressing the dual sensor R-GECO1.2-NLS/G-GECO1.2-NES. Timestamps are min:s. Scale bar represents 10 μm. **b** Normalised fluorescence intensity over time measured in the R-GECO1.2 (red) and G-GECO1.2 (blue) channels. **c** Percentage of plants that displayed cell autonomous nuclear Ca^2+^ spikes during root growth in Col-0, *dmi1-1*, and *dmi1-2* over 1 h and half of imaging. Numbers in bars represent the total of plants imaged (*n*) (*Χ*^2^ test, different letters represent *p* < 0.05). **d** Number of Ca^2+^ spikes per plants per hour in Col-0, *dmi1-1*, and *dmi1-2* within 200 μm of meristem length. No multiple spikes per cell were observed. Values are means ± s.e.m. Numbers in bars represent the total of plants imaged (*n*). n.s. not significant, **p < 0.01 (one-way ANOVA with Dunnet’s multiple comparison test vs. Col-0). **e** Measure of the primary root growth rate (cm) in function of cellular nuclear Ca^2+^ spike frequency. The growth rate was monitored at days 3 and 5 after germination and the Ca^2+^ signals monitored at day 6. The slope is calculated based on all points. **f** Representative nuclear Ca^2+^ trace in Col-0 and *dmi1-1*. Signals were aligned by their maxima. **g** Rise and fall times of the Ca^2+^ recorded in Col-0, *dmi1-1*, and *dmi1-2*. Values are means ± s.d. (one-way ANOVA with Bonferroni’s multiple comparison test, different letters represent *p* < 0.001 for both rise and fall times)

In root legume symbioses, the ion channels DMI1 and CNGC15 are required for the generation of nuclear-localised Ca^2+^ oscillations^7^. Once activated, they mediate the release of Ca^2+^ from the lumen of the nuclear/endoplasmic reticulum (ER)^7,14^. Our results demonstrate that AtDMI1 modulates root apical meristem development and growth in a non-symbiotic context. To investigate whether specific nuclear Ca^2+^ signals occur during root growth, we used a wild-type transgenic line expressing a dual-localised fluorescent Ca^2+^ sensor (Nuclear-localised Red Cytoplasmic-localised Green-GECO1.2; NRCG-GECO1.2) that allows simultaneous recording of cytosolic and nuclear Ca^2+^ signals^9^. We monitored the Ca^2+^ changes at the cellular level in the meristem and elongation zones of 5-day-old growing roots (Supplementary Fig. 8a, Supplementary Movies 1 and 2). Nuclear- and cytosolic-localised Ca^2+^ signals were observed in the meristem and elongation zones of growing wild-type roots, with a higher percentage of cells spiking in the meristematic zone (Supplementary Fig. 8b). Spatial and temporal analyses of these responses revealed that the Ca^2+^ release occurs first in the nucleus and subsequently extends to the cytosol (Fig. 2a, b and Supplementary Movie 2). Analysis of the spike shape using unbiased computational methods (Supplementary Fig. 9) allowed the precise characterisation of the nuclear Ca^2+^ signals. The nuclear Ca^2+^ signature observed is thus defined by one single Ca^2+^ spike with a mean duration of 39.5 s, a mean rise time of 15.4 s, and mean fall time of 24.1 s (Fig. 2f). The nuclear Ca^2+^ signature in the meristematic cell is identical to the one in the elongation zone, with one single spike per cell responding. An average of five cells responding is observed over 1 h of live imaging in wild-type meristems (Fig. 2d). These results demonstrate that specific nuclear Ca^2+^ signals occur during root growth in the root apical meristem.

### AtDMI1 modulates nuclear Ca^2+^ in root apical meristem

MtDMI1 has been shown to permeate cations and thought to provide counter-ion currents that facilitate Ca^2+^ release mediated by MtCNGC15s. Similarly, the mammalian trimeric intracellular cation channel (TRIC) couples with a Ca^2+^ channel to mediate Ca^2+^ release from the ER^15^. Knocking down of TRIC channels is sufficient to compromise Ca^2+^ release and notably to extend the duration of Ca^2+^ release leading to cardiac failure^15^. Thus overexpressing or removing a counter-ion channel can be sufficient to modulate the Ca^2+^ release. To assess whether DMI1 can modulate the nuclear Ca^2+^ release during root growth, we analysed the transgenic lines *dmi1-2* and *dmi1-1* expressing NRCG-GECO1.2 (Fig. 2c–f). In *dmi1-2* mutants, the percentage of plants where detectable nuclear Ca^2+^ spikes could be observed, as well as the frequency of spikes per plant meristem, were decreased in comparison to the wild type (Fig. 2c, d), suggesting that overexpression of *DMI1* impaired the activation of the nuclear Ca^2+^ channel. In contrast, in the knockdown *DMI1* mutant, *dmi1-1*, the frequency of nuclear Ca^2+^ release is unchanged, but the Ca^2+^ signature itself is impaired with an observed increase in both the rise and fall times of the Ca^2+^ release (Fig. 2c, f, g). Thus a modulation of the Ca^2+^ signature by increasing the duration of Ca^2+^ release is associated with longer expanded cells and thus longer roots. In contrast, reducing the growth-induced nuclear Ca^2+^ without altering the Ca^2+^ signature correlates with a shorter root meristem. This further suggests that the spike frequency is correlated with primary root growth rate. To challenge this hypothesis, we monitored the primary root growth over 5 days and analysed the frequency of cells generating nuclear Ca^2+^ release at the sixth day. Within roots responding in wild type and *dmi1-2*, the frequency of cells generating nuclear Ca^2+^ releases correlates with the primary root growth rate such that roots with the highest nuclear Ca^2+^ release frequency have the highest growth rate (Fig. 2e). These results demonstrate that AtDMI1 can modulate the nuclear Ca^2+^ signatures associated with primary root growth in Arabidopsis.

### AtDMI1 regulates auxin homoeostasis

Root meristem activity is controlled by the antagonist action of two main plant hormones, auxin and cytokinin, which are differentially distributed within the root apical meristem^16^. Notably, an auxin gradient that peaks at the quiescent centre and gradually decreases shootward is essential to maintain the balance of meristematic cell division and elongation^17^. To investigate whether nuclear Ca^2+^ spikes and auxin signalling activity colocalised, we investigated the spatial repartitioning of the Ca^2+^ signatures observed. In wild type, 80% of the nuclear Ca^2+^ signals were observed in meristematic cells (Supplementary Fig. 8b). Interestingly, in *dmi1-1* plants, which exhibited longer cells in the elongation zone, significantly more cells were spiking in the meristem as opposed to the elongation zone, in comparison to *dmi1-2* (Supplementary Fig. 8b). These results suggest that the nuclear Ca^2+^ release might follow the auxin distribution pattern in the root meristem, with high auxin levels correlating with more cells spiking and longer duration of release and lower auxin levels with fewer cells spiking. If this hypothesis is correct, one would expect to have higher auxin levels in the meristem of *dmi1-1* plants and the opposite in *dmi1-2* plants. To test this hypothesis, we investigated whether the distribution of endogenous auxin signalling was altered in these mutants in the root tip and meristem. We thus generated *dmi1-1* and *dmi1-2* lines expressing the DII-VENUS^18^ and DR5-GFP^19^. DII-VENUS is degraded in response to auxin signalling input and fluorescence inversely correlates with endogenous auxin levels, while DR5-GFP fluorescence reports transcriptional output of the auxin signalling pathway. DII-VENUS fluorescence intensity in the root tip and meristem was significantly reduced in *dmi1-1* roots and significantly increased in *dmi1-2* (Supplementary Figs. 10 and 11). Conversely, DR5-GFP fluorescence was significantly increased in *dmi1-1* root tips and reduced in *dmi1-2* roots (Supplementary Fig. 10). These results suggest that auxin accumulation and signalling in the root tip and meristem are higher in *dmi1-1* and weaker in *dmi1-2*. These results further suggest that the defect in Ca^2+^ signatures correlates with auxin distribution in the root meristem, with an increased duration of nuclear Ca^2+^ spikes associated with more auxin and a reduced frequency of signal occurrence with less auxin.

Auxin distribution in roots is tightly regulated by polar auxin transport and posttranscriptional and translational modifications^20,21^. To understand whether the defect in the nuclear Ca^2+^ signatures, which correlates with shorter or longer roots, modulates auxin homoeostasis via transcriptional regulation, we analysed the expression level of auxin-related genes in 6-day-old roots (Supplementary Fig. 12). RT-qPCR analyses revealed that mutants that have a defect in the number of cells spiking, and thus have shorter roots, display an increase in the expression of genes required for auxin biosynthesis, transport, and signalling (Supplementary Fig. 12). In contrast, longer spike duration, and thus longer elongating cells, correlates with a reduction in the expression of *PLETHORA1*, required for cell elongation^22^. This result demonstrates that differences in nuclear Ca^2+^ signature are correlated not only to longer or shorter roots but also to the differential expression of auxin-related genes. It further suggests that nuclear Ca^2+^ does contribute to fine-tune auxin homoeostasis.

### Increased auxin level rescue *dmi1-1* Ca^2+^ signature

**Fig. 3:**
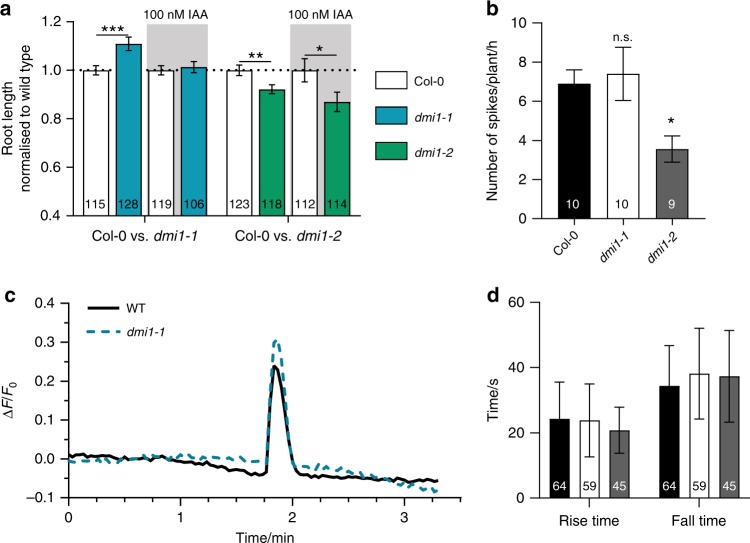
Auxin complements the root length and Ca^2+^ signature phenotype of *dmi1-1*. **a** Primary root length quantification of wild type (Col-0), *dmi1-1*, and *dmi1-2* grown in the absence or presence of 100 nM of indole-3-acetic acid (IAA) 6 days after germination (dag). The data represent three biological replicates. Values were normalised to wild type grown under the same conditions and represent means ± s.e.m. Numbers in bars denote sample size (*n*). *p < 0.05, **p < 0.01, ***p < 0.001 (two-tailed *t* test with a prior *F*-test for homoscedasticity). **b** Number of Ca^2+^ spikes per plant during 1 h of imaging at 2 h after gravitational stimulus in Col-0, *dmi1-1*, and *dmi1-2* bent side (high auxin), 5 dag. Values are means ± s.e.m. Numbers in bars represent the total of plants imaged (*n*). n.s. not significant, *p < 0.05 (one-way ANOVA with Dunnet’s multiple comparison test vs. Col-0). **c** Representative nuclear Ca^2+^ trace recorded in the bent side (high auxin) of Col-0 and *dmi1-1*. Signals were aligned by their maxima. **d** Rise and fall times of the Ca^2+^ spikes detected in the bent side (high auxin) of Col-0, *dmi1-1*, and *dmi1-2*. Values are means ± s.d. No statistical differences were found (one-way ANOVA with Bonferroni’s multiple comparison test)

We thus hypothesised that auxin homoeostasis could either regulate or be regulated by nuclear Ca^2+^ signalling. To test these hypotheses, we first grew the *dmi1-1* and *dmi1-2* mutants on media containing auxin or synthetic auxin at 100 and 1 nM. We observed that the *dmi1-2* mutant phenotype could not be rescued, unlike *dmi1-1*, which recovered the wild-type root length in all conditions tested (Fig. 3a, Supplementary Figs. 13 and 14). These results suggest that overexpressing DMI1 can block the auxin signalling correlated with nuclear Ca^2+^ signals. In contrast, the absence of DMI1 can be rescued, suggesting that the effect of auxin can restore normal nuclear Ca^2+^ signals in *dmi1-1*. To investigate the influence of auxin on the nuclear Ca^2+^ signal, we recorded the nuclear Ca^2+^ released in the root grown on auxin-containing media (Supplementary Fig. 15). In correlation with the root growth phenotype, auxin did not rescue the Ca^2+^ signal frequency defect of *dmi1-2*, but the Ca^2+^ signature of *dmi1-1*, characterised by an increased spike duration, reverted to the duration of a wild-type Ca^2+^ signal (Supplementary Fig. 15).

**Fig. 4:**
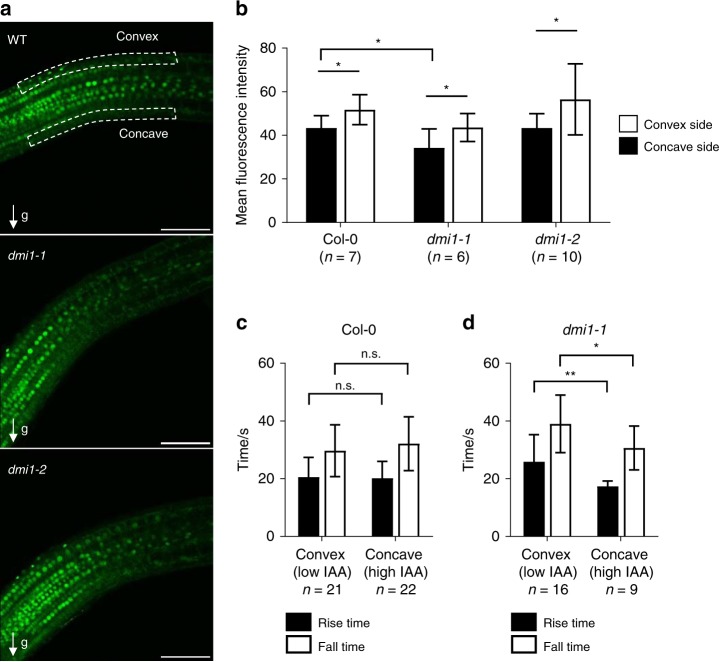
Analyses of auxin abundance and calcium spikes in the concave and convex side of bent roots. **a** Representative images of bent roots of Col-0, *dmi1-1*, and *dmi1-2* plants expressing DII-VENUS 6 days after germination and 90 min after a gravity stimulus (g) of 90 degrees. Dashed lines delimit area used for quantification in the concave (high auxin) and convex (low auxin) sides. Scale bars represent 100 µm. **b** Mean fluorescence intensity of the DII-VENUS signals in Col-0, *dmi1-1*, and *dmi1-2*. Intensity was averaged across the concave and convex delimited areas (marked in **a**). The total area was the same for each plant. Values are means ± s.d. The number of plants imaged (sample size) is indicated (*n*). **c**, **d** Rise and fall time in seconds (s) of calcium spikes recorded in the convex (low auxin) and concave (high auxin) areas in wild-type (**c**) and *dmi1-1* (**d**) roots. Values are means ± s.d. Sample size is indicated (number of spikes, *n*). n.s. not significant, *p < 0.05, **p < 0.01 (two-tailed *t* test with a prior *F*-test for homoscedasticity). The data represent two biological replicates

To confirm these results under physiological conditions, we increased endogenous auxin concentration locally by shifting the gravity vector^23^. Rotating growing roots by 90 degrees causes a shift in the flow of auxin through the root, with an increase of auxin accumulation in the underside of the root. A higher auxin accumulation on the lower side inhibits cell elongation, causing the root to bend^24^. It should be noted that *dmi1* mutant alleles are not defective in bending response (Supplementary Fig. 16). DII-VENUS fluorescence intensity analyses of the concave and convex side of the wild type, *dmi1-1*, and *dmi1-2* confirmed that endogenous auxin levels are significantly higher in the concave side of the bent root in comparison to the convex side within each genotype (Fig. 4a, b). In addition, the auxin level in the concave side of *dmi1-1* is significantly higher than in the wild-type concave side (Fig. 4b). We thus recorded nuclear Ca^2+^ signals in the concave sides after rotating the plants by 90 degrees. In correlation with the growth on auxin-containing media, increasing the endogenous auxin concentration did not rescue the Ca^2+^ signal frequency defect of *dmi1-2* (Fig. 3b), but the Ca^2+^ signature of *dmi1-1* reverted to the duration of a wild-type Ca^2+^ signal (Fig. 3c, d). In addition, comparative analyses of the Ca^2+^ spike duration between the concave and convex side within *dmi1-1* bent roots confirmed that the reversion to a wild-type Ca^2+^ signal is specifically associated with a significant auxin accumulation in the concave side of *dmi1-1* (Fig. 4b, d). This demonstrates that auxin homoeostasis does modulate nuclear Ca^2+^ signals. It further suggests that, in the absence of the counter-ion channel DMI1, a sufficient increase in auxin concentration may contribute to the activation of an alternative counter-ion channel, which allows the physiological release of Ca^2+^.

## Discussion

In this study, with the use of a dual-localised fluorescent Ca^2+^ sensor, we identified a novel nuclear-localised Ca^2+^ release occurring during root apical meristem growth. We showed that the growth induced-nuclear Ca^2+^ release is modulated by AtDMI1 and is associated with differences in primary root growth (Supplementary Fig. 17). We further demonstrate that modulating the ion channel genetically changes the nuclear Ca^2+^ signature, influences auxin homoeostasis, and leads to shorter or longer roots.

Previous work has highlighted that MtDMI1 and MtCNGC15 form a complex required to generate symbiotic factor-induced nuclear Ca^2+^ oscillation^7^. Upon activation, MtCNGC15 would assure Ca^2+^ release, and MtDMI1 the counter-ion movement of a positive charge^7^. Similarly to AtDMI1, we demonstrated that AtCNGC15 is expressed and localises to the nuclear envelope of *A. thaliana* root meristem cells, suggesting that they might function together to modulate the growth-induced nuclear Ca^2+^ release. In line with this hypothesis, bimolecular fluorescent complementation between AtCNGC15 and AtDMI1 in *A. thaliana* root indicates that AtCNGC15 and AtDMI1 reside in closed proximity at the nuclear envelope (Supplementary Fig. 18). These results suggest that AtCNGC15 and AtDMI1 might function together in root development, although further experiments are required to understand their dynamics. The overexpression of *AtDMI1* impairs the frequency of nuclear Ca^2+^ spikes, suggesting that, if AtDMI1 forms a complex with AtCNGC15, overexpressing AtDMI1 can block AtCNGC15 activation. On the other hand, removing DMI1 changes the duration of the nuclear Ca^2+^ release, which supports the role of DMI1 as a counter-ion channel. Therefore, our study highlights that modulating ion channels genetically can be an effective way to influence a specific Ca^2+^ signal and modulate a development process. Our data further support the postulate^25^ that the frequency and duration of a Ca^2+^ signal can encode stimulus-specific information that regulates cellular function.

In contrast to AtCNGC14, which is required for root bending and auxin-induced cytoplasmic Ca^2+^ release^26,27^, the *dmi1* mutant alleles do not have any defect in the root bending response. This demonstrates that AtDMI1 and AtCNGC14 have distinct functions in the context of auxin signalling. This observation further reinforces the postulate that spatial release of Ca^2+^ in specific cell types and cellular compartments plays a role in specifying the output of the signal transduction via the selective activation of spatially and temporally regulated Ca^2+^-binding proteins.

Interestingly, increasing the external or internal concentrations of auxin can rescue the absence of DMI1 by potentially activating an alternative counter-ion channel, which allows the physiological release of Ca^2+^. Thus auxin signalling can modulate nuclear Ca^2+^ signals in the absence of DMI1 (Supplementary Fig. 17). In addition, analyses of auxin-related gene expression and auxin repartition in the *dmi1* mutants demonstrate that DMI1 also regulates auxin homoeostasis. Whether DMI1-mediated changes in nuclear Ca^2+^ regulation are mechanistically directly related to the changes in auxin homoeostasis in root apical meristem growth is unknown. However, the spatial pattern of the nuclear Ca^2+^ release over the meristem and elongation zone, as well as the root phenotype of *dmi1-1* and *dmi1-2*, suggest that spatial regulation of specific components via posttranslational modification and/or expression regulation is likely involved. Thus our study also sheds light on nuclear Ca^2+^ signalling correlated with auxin signalling in the root apical meristem and potentially required to integrate growth-related signals to fine-tune auxin homoeostasis and sustain primary root growth.

## Methods

### Plant material and growth conditions

The SALK and SAIL lines (N814022: *dmi1-2*, N653269: *dmi1-1*, WisDsLox437E09: *cngc14-2*) and wild-type Col were purchased from the Nottingham Arabidopsis Stock Centre. Arabidopsis seeds were surface-sterilised in 1.5% bleach for 15 min, followed by 5 washes in sterile water, and the seeds were then plated in Murashige and Skoog (MS)/2-(*N*-morpholino)ethanesulfonic acid (MES), 1% sucrose, and 0.8% agar pH 5.8. For bending experiments, the seeds were directly sown on sterile slide cover with MS/MES medium described above with the following modification: 0.8% phytagel. For Ca^2+^ imaging, as only 2–3 plants could be analysed per day, plants were grown every 3 days until completion of the experiment. After 3–5 days at 4 °C, plates were moved to a growth cabinet (23 °C, 16‐h photoperiod, and 300 µmol m^−2^ s^−1^ light intensity).

### Genotyping

Homozygosity was assessed by PCR. Primers were designed using the SIGnAL T-DNA Primer Design algorithm (http://signal.salk.edu/tdnaprimers.2.html) for SALK and SAIL alleles (Supplementary Table 1).

### Golden gate cloning

Golden gate cloning followed the principles outlined in Engler et al.^28,29^. Level 0 modules were synthesised by Life Technologies™ (ThermoFisher Scientific) and assembled as described in Supplementary Table 2.

### Generation of Arabidopsis stable transgenic lines

Arabidopsis stable transgenic lines were generated using *Agrobacterium tumefaciens*-mediated gene transfer by inflorescence infiltration. Plants were grown for 4–5 weeks (T0 generation) prior to floral dipping, and seeds were collected and germinated under the appropriate selection in the T1 and T2 generations^30^. *dmi1-1* and *dmi1-2* plants expressing DII-VENUS or DR5-GFP were obtained by manual hand-pollination of individual lines with Columbia DII-Venus and DR5-GFP described previously^18,19^.

### Nodulation and mycorrhization complementation assays

The Golden gate constructs pAtUBI10:GFP and pATUBI10:gAtCNGC15:GFP (Supplementary Table S2) were expressed in *M. truncatula* roots using *Agrobacterium rhizogenes*-mediated gene transfer performed as previously described^7^. The *A. rhizogenes* strain AR1193 was used. Each construct expresses the fluorescent marker mCherry fused to a nuclear exclusion signal as a plant marker to facilitate the selection of transformed roots by fluorescence microscopy. To monitor AM root length colonisation, plants were grown in Terragreen/Sand (Oil-Dri Company, Wisbech, UK) and inoculated with *Rhizophagus irregularis* (Endorize; Agrauxine, France) to the ratio 5:5:1 (Terragreen/Sand/Spores). The fungal structures were stained in acidic ink as follows: roots were cleared in 10% KOH 15 min at 95 °C, washed 3 times in water, and subsequently stained in acidic ink (5% ink, 5% acetic acid) for 4 min at 95 °C. The AM root length colonisation was quantified as previously described^7^. For nodulation assays, 1-week-old plants were grown in Terragreen/Sand (Oil-Dri Company, Wisbech, UK) to a ratio (1:1) and inoculated with *Sinorhizobium meliloti* 2011 (OD_600_ = 0.001). Nodules were scored as indicated.

### Quantification of gene expression

Total RNA was extracted using the RNeasy® Plant Mini Kit (QIAGEN) according to the instructions of the manufacturer. A subsequent step of on-column DNase digestion was included. Concentration and purity were determined by spectrophotometry (A260/280 and A260/230 ratios) and integrity was confirmed by gel electrophoresis (1% (w/v) agarose). cDNA was obtained from 500 to 2000 ng of RNA using the SuperScript™ III Reverse Transcriptase (Invitrogen™) according to the instructions of the manufacturer. qPCR was performed using a CFX96 Touch™ Real-Time PCR Detection System (BIO-RAD) with SYBR® Green JumpStart™ Taq ReadyMix™ (Sigma-Aldrich). For each primer set, amplification efficiency (*E*) was first determined through a cDNA dilution series. The qPCR were set up with an initial denaturation step (2 min at 95 °C), followed by 40 cycles of amplification and quantification (15 s at 95 °C; 15 s at 56 °C or 58 °C, and 30 s at 72 °C, with a single fluorescence measurement). A melt curve was also generated to verify the specificity of the amplification reaction (50–95 °C, with a fluorescence measurement every 0.5 °C). Calculation of the normalised expression and fold change ratio was performed using the mathematical model described previously^31^. Primers used for qPCR are listed in Supplementary Table S1.

### Root phenotype measurements

Seedlings were grown vertically on MS–MES plates as follows: 6 seedlings of control genotype and 6 seedlings of test genotype, per plate, and photographed at days 6 and 12 for the primary root length phenotyping; 4 seedlings of control genotype and 4 seedlings of test genotype, per plate, and photographed at days 3 and 5 for the growth rate followed by Ca^2+^ imaging; 6 seedlings per plate for root bending experiment and photographed at day 6 after bending. Root length and bending angle were quantified using ImageJ 1.48v (NeuronJ plugin, 1.4.3v).

### Characterisation of the root meristem and transition zone

Seedlings were grown vertically on MS–MES plates (6 seedlings of control genotype and 6 seedlings of test genotype, per plate) and analysed at days 6 or 12. Seedlings were fixed in 50% methanol 10% acetic acid, for at least 24 h at 4 °C. Samples were then washed with dH_2_O, washed with 20 mM phosphate buffer, and incubated overnight at 37 °C in a α-amylase suspended in 20 mM phosphate buffer. Samples were then washed with dH_2_O, incubated in 1% periodic acid at room temperature for 40 min, washed with dH_2_O, incubated in freshly prepared Schiff’s reagent (100 mM sodium metabisulfite; 0.15 N HCl; 20 µg mL^−1^ propidium iodide) for 30 min or until roots had acquired a pink colour, washed with dH_2_O, and incubated overnight at room temperature in chloral hydrate:glycerol:water (8 g:1 mL:2 mL). Samples were then mounted in chloral hydrate and imaged using a Zeiss LSM 780 confocal microscope (Carl Zeiss). Cell counting and cell length measuring were performed using ImageJ 1.48v (Cell-o-Tape plugin, 0.7.7v)^32^. This macro was used to count and measure the cells along the cortex file. The first rapidly elongating cortex cell was identified using the Cell-o-Tape plugin and confirmed by visual inspection.

### Histochemical GUS staining of Arabidopsis plants

Seedlings were fixed in cold 90% acetone for at least 30 min at 4 °C. Acetone was removed, and the material was washed twice with rinse solution (0.5 M Na_2_HPO_4_, 0.5 M NaH_2_PO_4_, 0.1 M K_3_Fe(CN)_6_, 0.1 M K_4_Fe(CN)_6_). The rinse solution was removed, and stain solution added (rinse solution complemented with 2 mM x-Gluc (5‐bromo‐4‐chloro‐3‐indolyl‐beta‐D‐glucuronide). Samples were then gently vacuum-infiltrated for 30 min and then incubated at 37 °C, in the dark, for 30 min or 24 h. Samples were then washed in water and cleared in chloral hydrate (8 g choral hydrate, 3 mL 100% glycerol, 1 mL dH_2_O) for at least 45 min, before mounting in chloral hydrate.

Embedding primary roots GUS stained for sectioning was performed using Technovit 7100 according to the manufacturer’s instruction (Electron Microscopy Science). Sectioning was performed using a Leica UC7 (Ultra-microtome). Images were obtained with a DM6000 microscope (Leica).

### Ca^2+^ imaging

Ca^2+^ imaging was performed using a Nikon ECLIPSE FN1 as described in Kelner et al.^13^. Seedlings were collected 5 days after germination and carefully placed in a small chamber made on a coverslip using high-vacuum grease (Dow Corning GMBH, Wiesbaden, Germany). The chamber was filled with a small volume of MS (50–100 µL) and closed with a smaller coverslip, covering the entire root. The seedling was then incubated at room temperature for at least 45 min before imaging. Images were collected every 2 or 3 s, for an average period of 1.5 h, focussed on the tip of the root upwards as indicated.

For Ca^2+^ imaging of gravistimulated roots, the plants were grown vertically on top of MS medium (0.8% phytagel) over cover slides. Two hours before imaging, a coverslip was gently put on top of the root and the plate, remaining vertical, was rotated 90 degrees. After 2 h of gravistimulation, the sample was imaged as described above, for 1 h. For all the Ca^2+^ imaging experiments, the number of biological replicates is *n*/3 as the number of plants recorded per day is 3.

### Ca^2+^ imaging analysis

For image processing, the following steps were conducted using ImageJ 1.48v: background subtraction, registration using MultiStackReg v1.45 (http://bradbusse.net/sciencedownloads.html), and application of a lookup table. Image data were obtained from processed images using Time Series Analyser V3_2 (https://imagej.nih.gov/ij/plugins/time-series.html). Normalised data sets (Δ*F*/*F*) were calculated as (*F* − *F*_0_)/*F*_0_, where *F*_0_ represent the average of the first 20 frames of baseline values. To analyse spike shape, an algorithm was designed to quantify the rise and fall times of the spikes observed during Ca^2+^ imaging. The script developed to analyse the fall and rise times of the Ca^2+^ spike is included as Supplementary Data 1 and from the online git repository gitlab at https://gitlab.com/rosscarter33/calcium_splike_analysis. Data were detrended by subtracting a polynomial fit to the raw fluorescence data. The detrended signal and the gradient of the detrended signal were used to identify the peak (maximum value of the detrended signal) and the beginning and end of the pulse, these being defined as the first point in time before and after the peak, respectively, where both the detrended signal and the gradient fall below a threshold. These values are then used to calculate the rise and fall times.

### Confocal laser scanning microscopy

Images of AtCNGC15-GFP and AtDMI1-GFP localisation and control, as well as DR5GFP and DIIVENUS, were collected using a Zeiss LSM780 confocal microscope with a ×40/1.2 water objective or a ×20/0.5 dry objective. Details of the excitation and emission wavelengths are as follows: GFP excitation wavelength 488 nm, emitted fluorescence collected at 500–550 nm. VENUS excitation wavelength 488 nm, emitted fluorescence collected at 505–530 nm.

### Fluorescence intensity quantification

For DII-VENUS and DR5-GFP, fluorescence intensity quantification were conducted using ImageJ 1.48 v.

### Statistical analyses

Statistical analyses were performed using GraphPad Prism version 5.00 for Windows (GraphPad Software, La Jolla, CA, USA, www.graphpad.com).

### Reporting summary

Further information on research design is available in the Nature Research Reporting Summary linked to this article.

## Supplementary information

Supplementary Information

Description of Additional Supplementary Files

Supplementary Movie 1

Supplementary Movie 2

Supplementary Data 1

Reporting Summary

Source Data

## Data Availability

Source data are provided for Figs. 1, 2, 3, and 4 and Supplementary Figs. 1, 2, 6, 7, 8, 10, 11, 12, 13, 14, 15, and 16. All other data are available from the corresponding author upon reasonable request.

## Code availability

The script developed to analyse the fall and rise times of the calcium spike is included as Supplementary Data 1 and from the online git repository gitlab at https://gitlab.com/rosscarter33/calcium_splike_analysis.
