# Supplementary material for: Nuclear calcium signatures are associated with root development
Source: Nat Commun. 2019 Oct 25;10:4865. doi: 10.1038/s41467-019-12845-8 (PMC6814746; doi:10.1038/s41467-019-12845-8)
Supplement: Supplementary file 1 — Supplementary Information [file 41467_2019_12845_MOESM1_ESM.pdf]

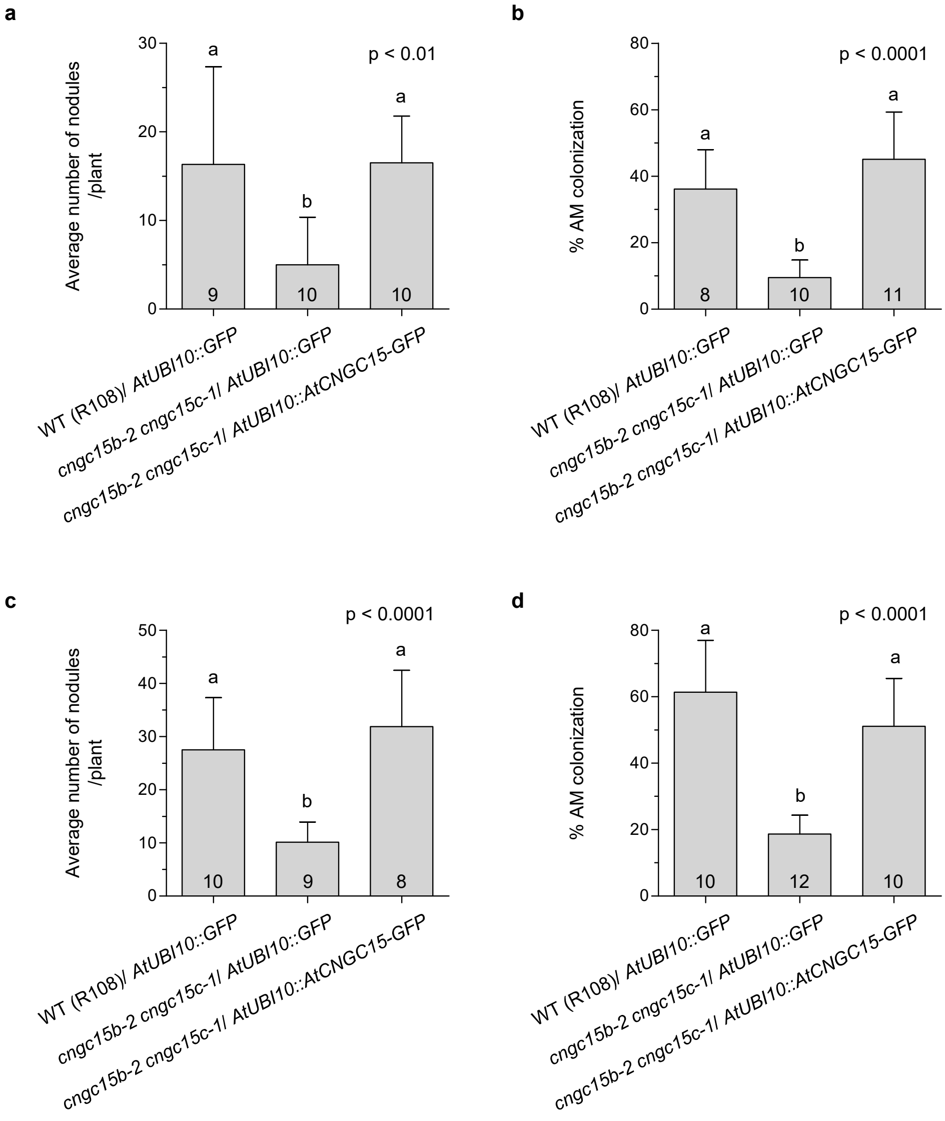

Supplementary Figure 1 | *AtCNGC15* rescues the symbiotic phenotypes of *M. truncatula cngc15b-2 cngc15c-1* double mutant.

(**a, c**) Complementation of the nodulation and (**b, d**) mycorrhization phenotypes of the *M. truncatula cngc15b-2 cngc15c-1* double mutant by expressing *AtCNGC15* fused to GFP, driven by the Arabidopsis *UBIQUITIN10* promoter. Nodulation was assayed 25 days after inoculation with *S. meliloti* strain 2011 and mycorrhization was assessed 5 weeks after inoculation with *R. irregularis*. **a** and **c** represent two independent replicates of the nodulation assay, **b** and **d** represent two independent replicates of the mycorrhization assay. Bar graphs show mean ± s. d.. Numbers in bars denote sample size (n). Different letters indicate statistical differences and p-values are inscribed (one-way ANOVA with Bonferroni's multiple comparison post-test).

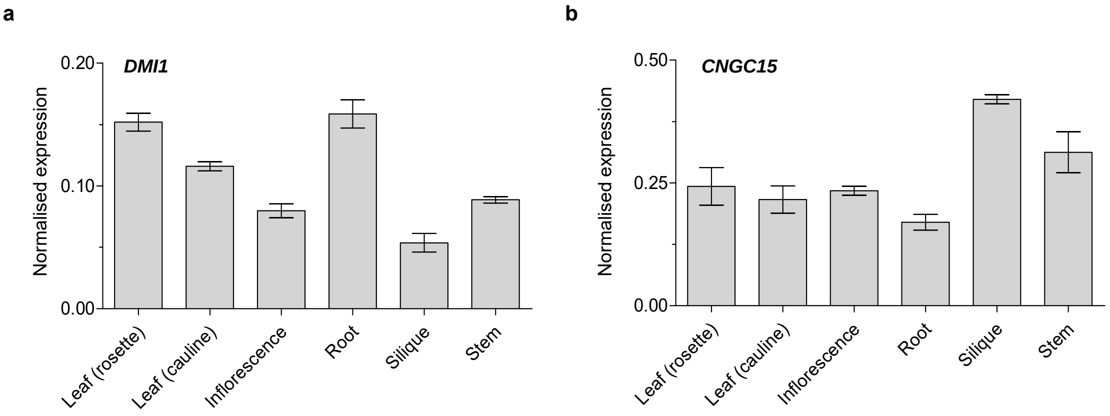

Supplementary Figure 2 | *DMI1* and *CNGC15* are expressed in *A. thaliana*.

Quantitative expression analyses of the transcript level of *DMI1* (**a**) and *CNGC15* (**b**) by RT-qPCR in rosette leaves, cauline leaves, inflorescences, siliques, stems (5-week-old plants) and roots (2-week-old) (n = 4). Expression was normalised to *UBOX* (*At5g15400*). Values are means ± s. e. m..

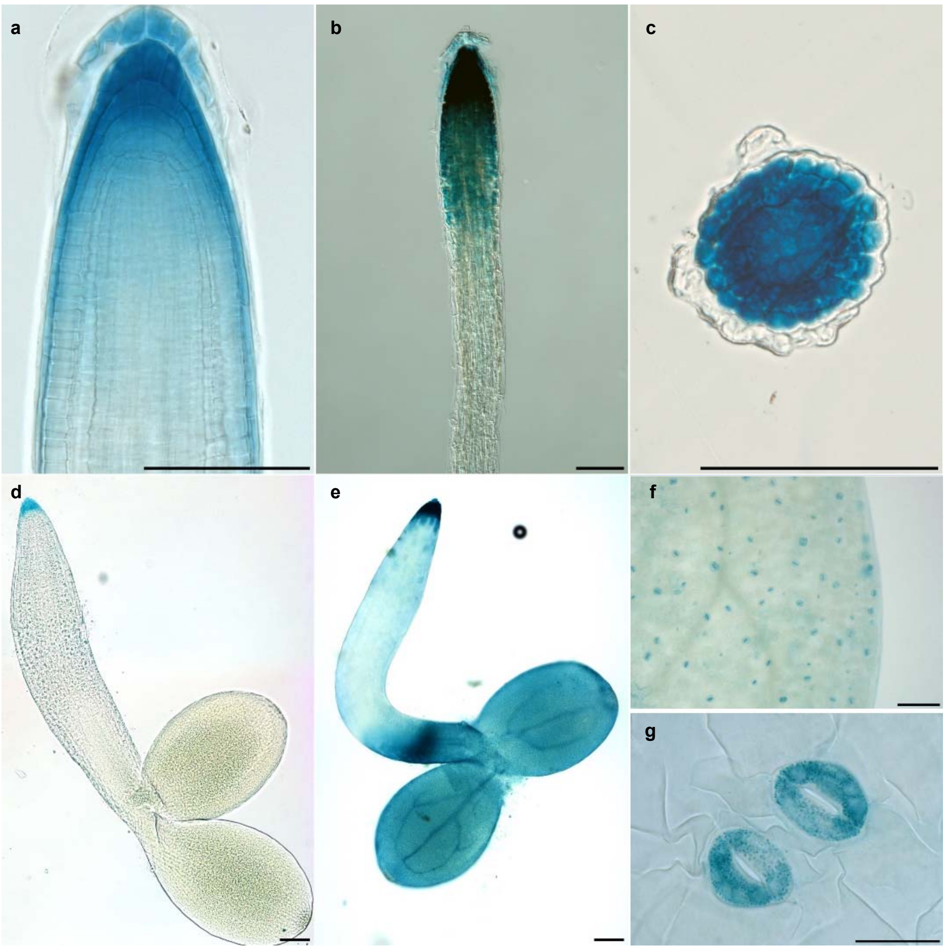

Supplementary Figure 3 | *CNGC15* is expressed in the root meristem.

a-d, GUS staining in *pAtCNGC15::GUS* seedlings 3 days after germination (**a**), 6 dag (**b, c**), 1 dag (**d**, **e**), and 5 dag (**f, g**). The reaction was incubated for 30 min (**a, d**), 1h (**b**, **c**) or 24 h (**e, f, g**). Scale bars represent 100 µm for all images, except **g**) where it represents 20 µm. *AtCNGC15* is strongly expressed in root apical meristem in germinated seedling (**d**, **e**) as well as developed seedlings (**a**, **b**, **c**) and in all cell layers of root apical meristem (**c**). (**c**) Transversal section of the root apical meristem above the root cap. In addition, *AtCNGC15* is constitutively expressed in cotyledons (**e**) but specifically in guard cell in mature leaves (**f**, **g**).

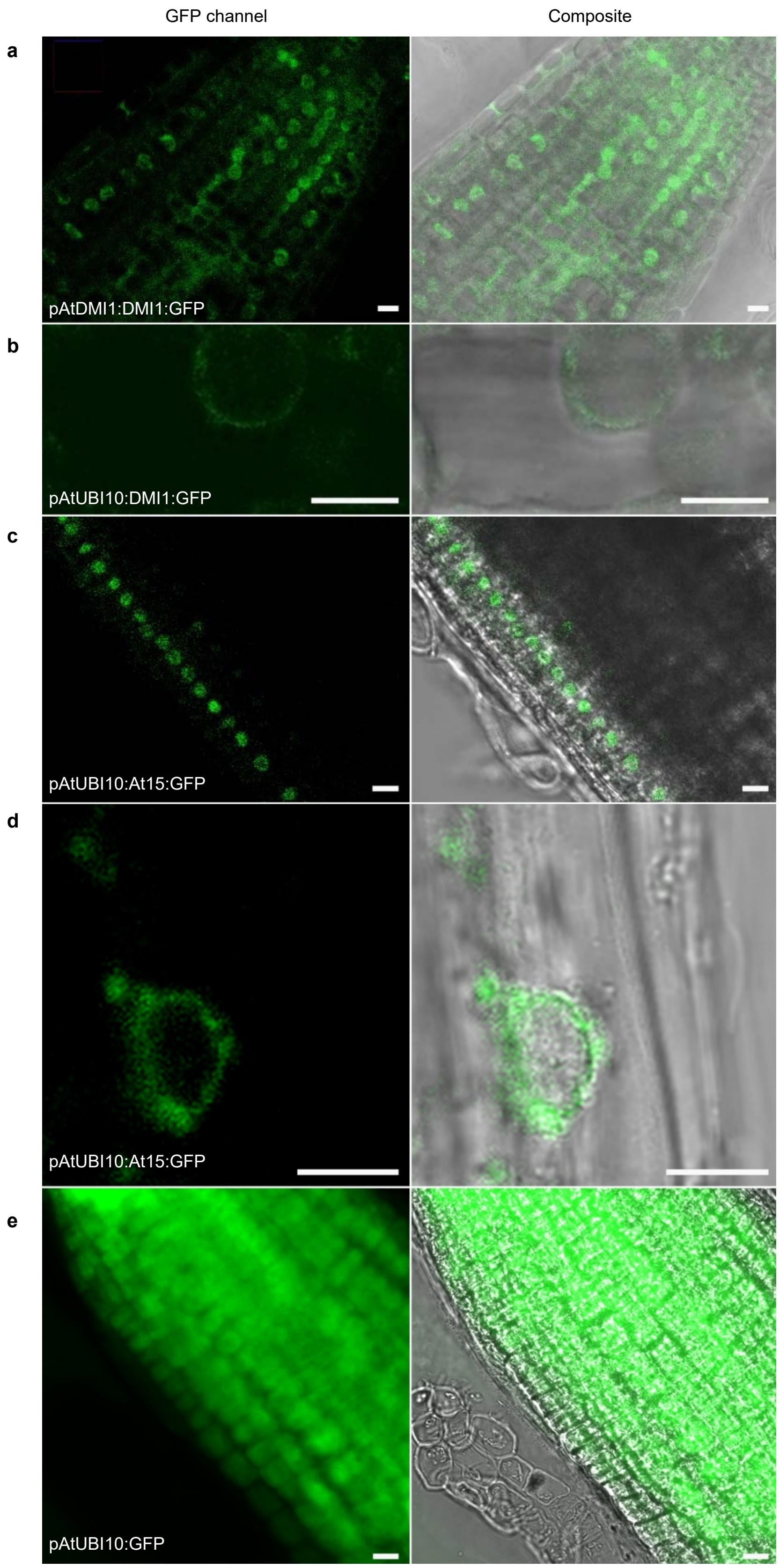

Supplementary Figure 4 | *DMI1 and* CNGC15 are localised to the nuclear envelope.

Laser scanning confocal microscopy images of 6 day-old *A. thaliana* root meristems expressing (**a**) *pAtDMI1::gDMI1-GFP,* (**b**) *pAtUBI10::cDMI1-GFP*, (**c**) *pAtUBI10::gCNGC15-GFP*, (**d**) *pAtUBI10::gCNGC15-GFP*, and (**e**) *pAtUBI10::GFP*. Green fluorescent protein (GFP) detection on the right panel, and overlay picture of the green channel and bright field on the left panel. Scale bars represent 10 µm.

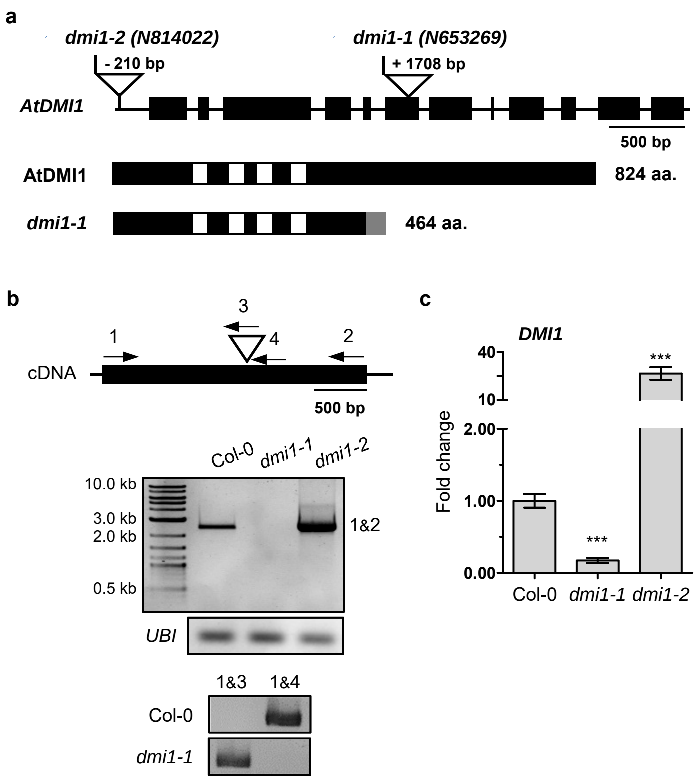

Supplementary Figure 5 | Identification of *DMI1* mutants in *A. thaliana*.

a, b, Positions of the T-DNA insertions in *DMI1*, confirmed by PCR and sequencing. Exons are represented by black boxes and introns by black lines (top). The predicted structure of the wild-type amino acid sequences is represented, with transmembrane domains in white and the modified C-terminus derived from the T-DNA in grey (bottom). **b**, Expression analysis using RT-PCR in Col-0, *dmi1-1* and *dmi1-2*. The position of the primers on the cDNA is indicated by arrows (top). **c**, Quantitative expression analysis of the transcript level of *DMI1* by RT-qPCR in root samples 6 days after germination (n = 4, pools of approximately 400 plants). Expression was normalised to *UBOX* (*At5g15400*) and presented as fold change over control (Col-0). Values are means ± s. d. *** p < 0.001 (two-tailed t-test with a prior F-test for homoscedasticity).

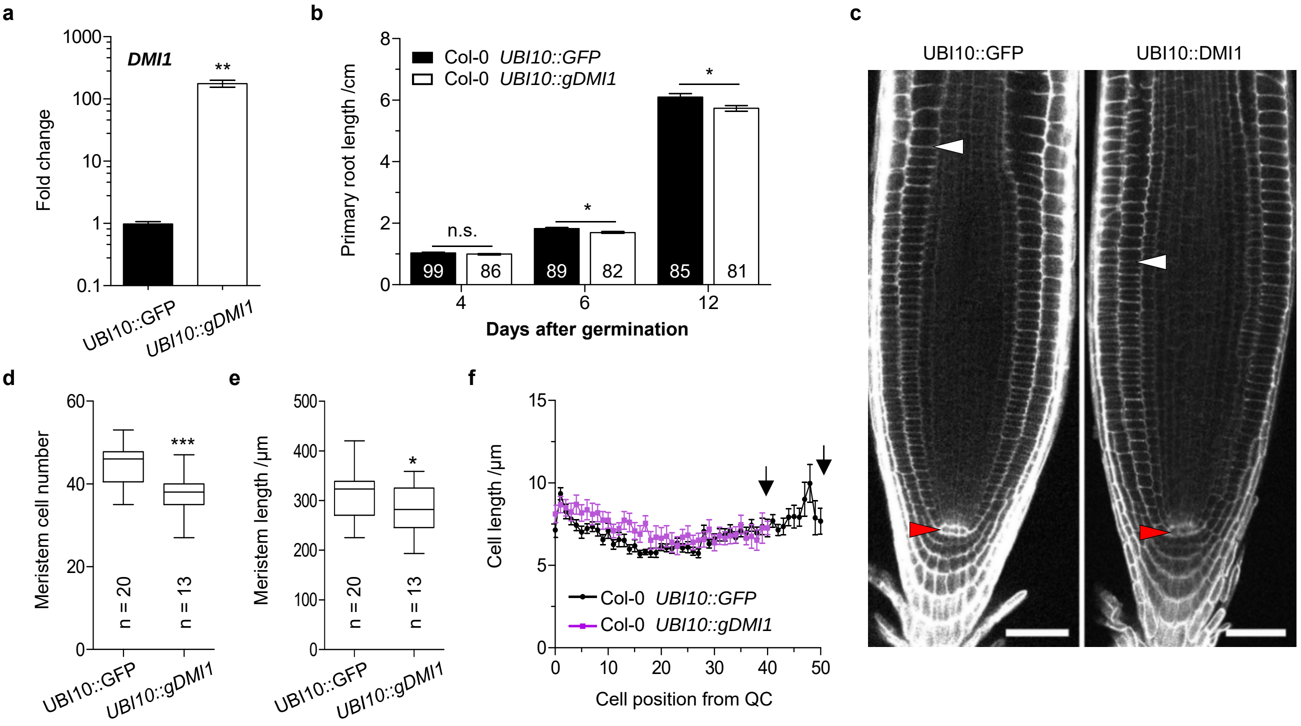

Supplementary Figure 6 | Overexpression of *DMI1* recapitulates the *dmi1-2* phenotype.

a, **b**, Quantification of *DMI1* transcript (n = 3 or 4, pools of 40-60 plants) (**a**) and primary root length time course (**b**) in root samples of control (Col-0 *UBI10::GFP*) and a *DMI1* overexpression line (Col-0 *UBI10::gDMI1*). Expression was normalised to *UBOX* (*At5g15400*). Values show mean ± s. d. **c**, Longitudinal view of the root meristem of control (Col-0 *UBI10::GFP*) and the *DMI1* overexpression line (Col-0 *UBI10::gDMI1*). White and red triangles mark the first elongated cortex cell and the quiescent centre (QC), respectively. Scale bars represent 50 µm. The data represent three biological replicates. **d**, **e**, **f**, Root meristem cell number (**d**), root meristem length (**e**), and cell length over cell position from the QC to the last meristematic cell (black arrows) (**f**) of control (Col-0 *UBI10::GFP*) and *DMI1* overexpression line (Col-0 *UBI10::gDMI1*). Meristem cell number, length, and cell length profile were quantified 12 days after germination. The data represent two biological replicates. Black arrows in **f** mark the last meristematic cell. Box and whisker plots show median, minimum and maximum. Bar graphs show mean ± s. e. m.. Numbers in bars denote sample size (n). n.s. not significant, * $p < 0.05$, ** $p < 0.01$, *** $p < 0.001$ (two-tailed t-test with a prior F-test for homoscedasticity).

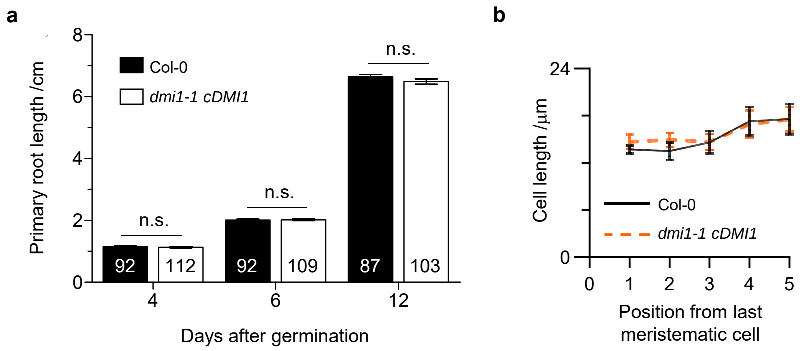

Supplementary Figure 7 | Complementation of *dmi1-1* phenotype.

a, Primary root length time course in root samples of control (Col-0 *UBI10::GFP*, black bars) and a *dmi1-1* complementation line (*dmi1-1 UBI10::cDMI1:GFP*, white bars). Values in bars are means ± s. e. m.. n.s. not significant. The data represent three biological replicates. **b**, Cell length over cell position from the first rapidly elongated cortex cell of Col-0 and a *dmi1-1* complementation line (*dmi1-1 UBI10::cDMI1:GFP*) quantified at six days after germination (at each cell position $n \geq 9$ for each population). The data represent two biological replicates. No statistical differences between both were found at each cell position (from 1 to 5). Two-tailed t-test with a prior F-test for homoscedasticity.

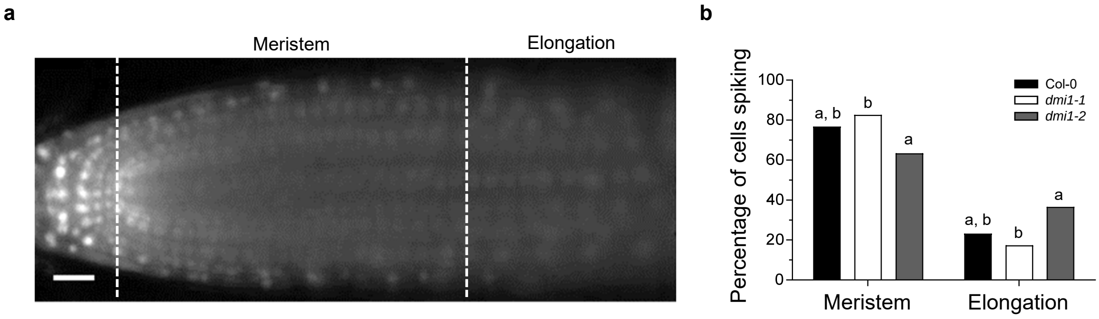

Supplementary Figure 8 | Growth-induced Ca^{2+} signals occur in the meristematic cells and elongation zone.

a, Representative image of a root expressing the dual Ca^{2+} reporter NRCG-GECO1.2 five days after germination (dag) indicating the meristem and elongation zone where the calcium signals were recorded. The dual Ca^{2+} reporter is composed of the red Ca^{2+} sensor R-GECO1.2 (shown) with a nuclear localisation signal, and the green Ca^{2+} G-GECO1.2 with a nuclear exclusion signal. Scale bar represents 25 µm. **b**, Percentage of Ca^{2+} signals localised to the meristem and elongation zones in Col-0 (n = 69), *dmi1-1* (n = 69), and *dmi1-2* (n = 52) expressing the dual Ca^{2+} reporter NRCG-GECO1.2 at 5 dag (χ^2 test, different letters indicate $p < 0.05$).

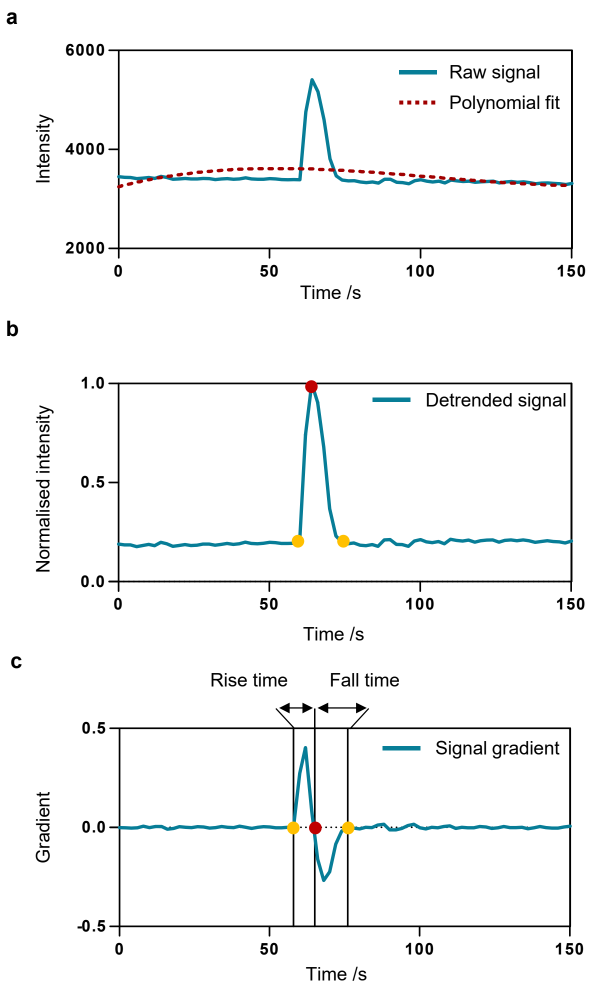

Supplementary Figure 9 | Calculation of rise and fall times.

a-c, The raw signal (intensity over time) is detrended by subtracting a polynomial fit (**a**). Signal maximum is the maximum value in the detrended data (**b**, red circles). The beginning and end of the signal time points (yellow circles) are determined by identifying the point in which the detrended signal and the signal gradient (**c**) fall below a defined threshold. Rise time is calculated as the difference between the times at peak maximum and the start of the peak, and fall time as the difference between times at the end of the peak and peak maximum.

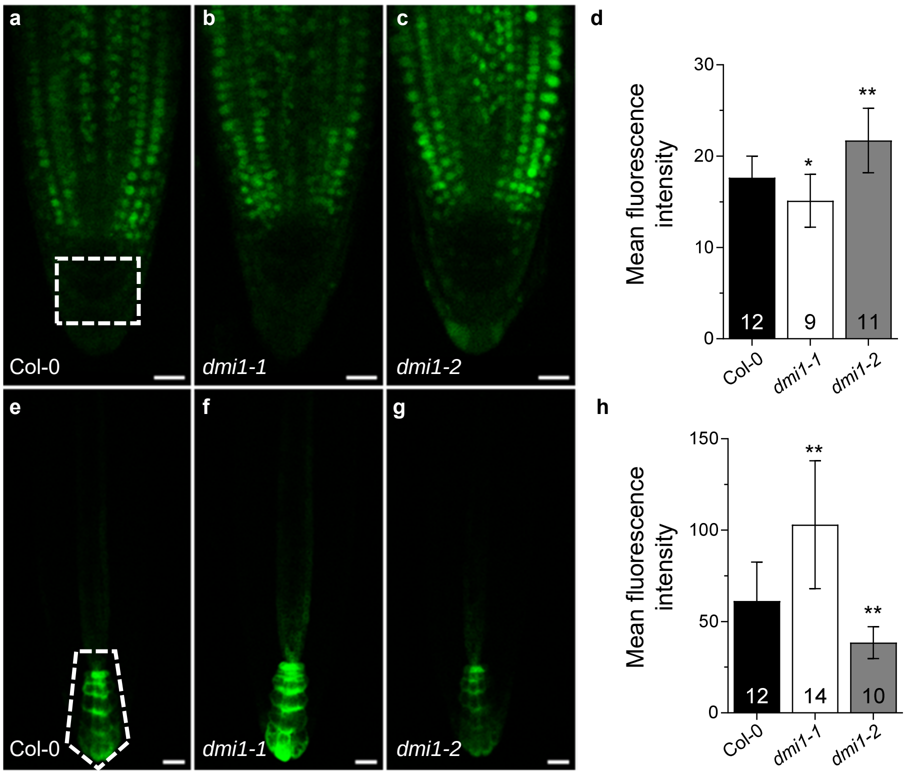

Supplementary Figure 10 | *dmi1* mutants have altered levels of auxin in the root columella.

Representative images of root tips of Col-0, *dmi1-1*, and *dmi1-2* plants expressing DII-VENUS (**a**-**c**) or DR5-GFP (**e**-**g**) six days after germination. Dashed lines delimit area used for quantification. Scale bars represent 20 µm. **d**, **h**, Mean fluorescence intensity of the DII-VENUS (**d**) or DR5-GFP (**h**) signals in Col-0, *dmi1-1*, and *dmi1-2*. Intensity was averaged across the xx axis for successive lines down the yy axis, within the delimited areas (marked in **a** and **f**). The total area was the same for each plant. Values are means ± s. d.. * $p < 0.05$, ** $p < 0.01$ (two-tailed t-test with a prior F-test for homoscedasticity).

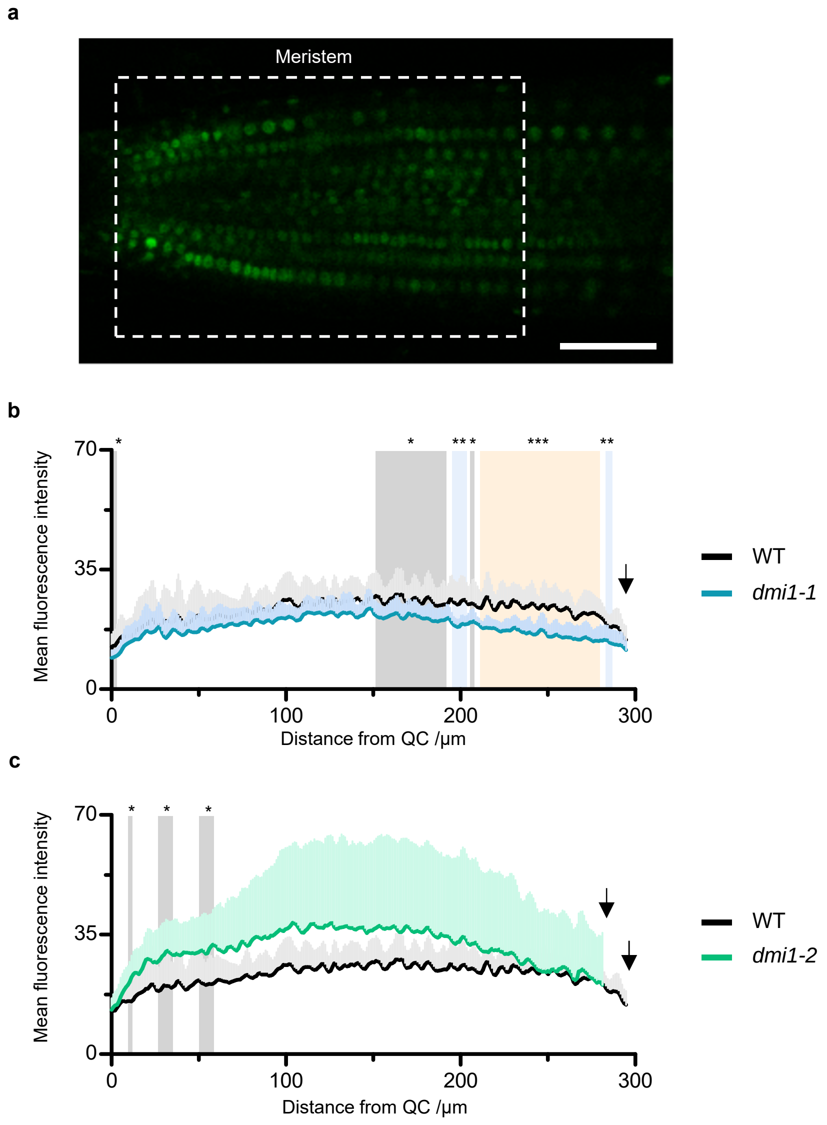

Supplementary Figure 11 | *dmi1* mutants have altered levels of auxin in the meristem.

a, Representative image of a Col-0 root expressing DII-VENUS 6 days after germination. Dashed lines delimit area used for quantification of the meristem. Scale bar represents 20 µm. **b-c,** Mean fluorescence intensity of the DII-VENUS signals in Col-0 (**b-c**), *dmi1-1* (**b**), and *dmi1-2* (**c**), in the meristem**.** Intensity was averaged across the xx axis for successive lines down the yy axis, within the delimited area (marked in **a**). The total area was the same for each plant. Values are means ± s. d. with sample size n = 13 (WT), n = 11 (*dmi1-1*), n = 10 (*dmi1-2*). Black arrows in **b-c** mark the last meristematic cell. Significant differences are marked by shaded areas and indicated with * ($p < 0.05$), ** ($p < 0.01$), or *** ($p < 0.001$), as determined by a two-tailed t-test with a prior F-test for homoscedasticity.

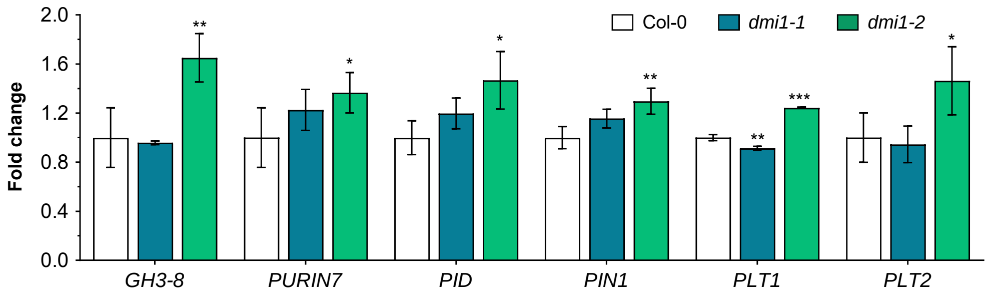

Supplementary Figure 12 | Transcription auxin marker genes in *dmi1* and *cngc15* mutants.

Quantitative expression analyses of the transcript level of auxin-related genes by qPCR in root samples of Col-0, *dmi1-1*, and *dmi1-2* (n = 3 or 4, each sample was a pool of approximately 400 plants), 6 days after germination. Expression was normalised to *UBOX* (*At5g15400*) and results are presented as fold change over wild type (Col-0). Values are means ± s. d.. * $p < 0.05$, ** $p < 0.01$, *** $p < 0.001$ (two-tailed t-test with a prior F-test for homoscedasticity).

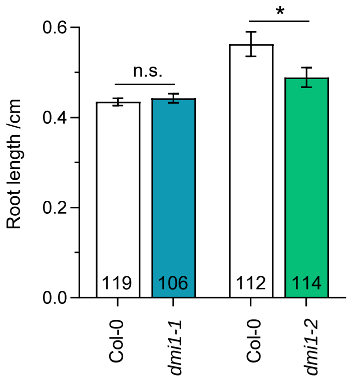

Supplementary Figure 13 | Auxin complements the root length phenotype of *dmi1-1*.

Primary root length quantification of wild type (Col-0), *dmi1-1*, and *dmi1-2*, grown in the presence of 100 nM of indole-3-acetic acid (IAA) 6 days after germination. Values represent means ± s. e. m.. Numbers in bars denote sample size (n). n.s. not significant, * $p < 0.05$ (two-tailed t-test with a prior F-test for homoscedasticity). The data represent three biological replicates.

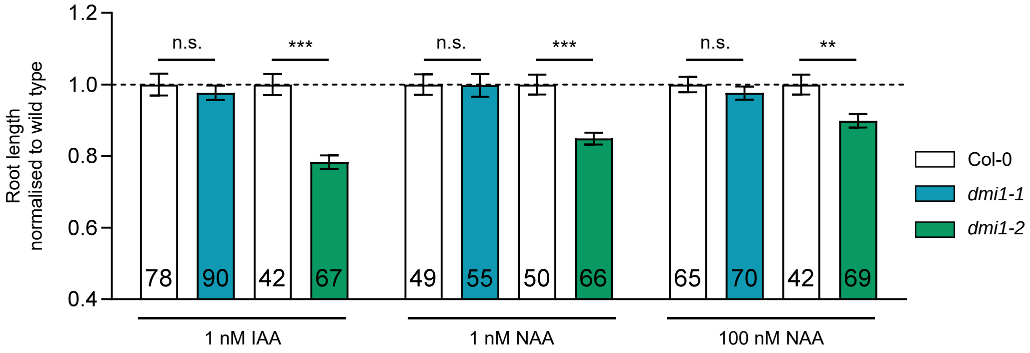

Supplementary Figure 14 | IAA and NAA complement the root length phenotype of *dmi1-1* mutant

Primary root length quantification 6 days after germination of wild type (Col-0), *dmi1-1*, and *dmi1-2* grown in the presence of 1 nM of indole-3-acetic acid (IAA), 1 nM of 1-aphthaleneacetic acid (NAA) or 100 nM NAA. Values were normalised to wild type grown under the same conditions and represent means ± s. e. m.. Numbers in bars denote sample size (n). n.s. not significant, ** $p < 0.01$, *** $p < 0.001$ (two-tailed t-test with a prior F-test for homoscedasticity).

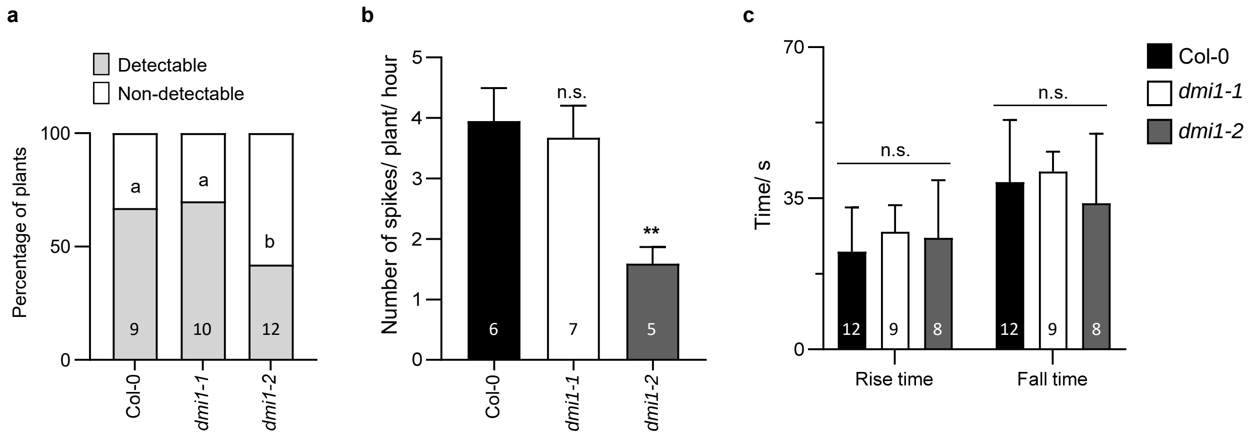

Supplementary Figure 15 | Growth on auxin –containing media rescue *dmi1-1* calcium signature

a, Percentage of plants that displayed cell autonomous nuclear Ca^{2+} spikes during root growth in Col-0, *dmi1-1* and *dmi1-2* over one hour of imaging. Numbers in bars represent the total of plants imaged (n) (X^2 test, different letters represent $p < 0.05$). **b**, Number of Ca^{2+} spikes per plants per hour in Col-0, *dmi1-1* and *dmi1-2.* Numbers in bars represent the total of plants imaged (n). Values are means ± s.e.m.. n.s. not significant, ** $p < 0.01$ (one-way ANOVA with Dunnet's multiple comparison test vs. Col-0). **c**, Rise and fall times of the Ca^{2+} recorded in Col-0, *dmi1-1* and *dmi1-2.* Values are means ± s.d.. No statistical differences (n.s.) were found (one-way ANOVA with Bonferroni's multiple comparison test). Analyses were performed on plant grown on 1 nM IAA, 6 days after germination.

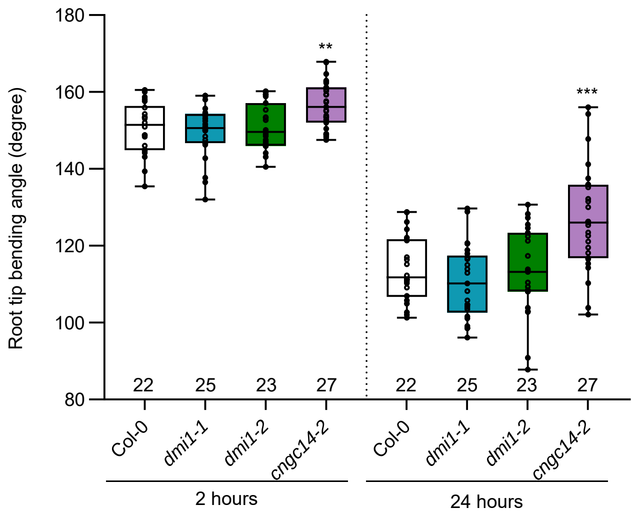

Supplementary Figure 16 | *dmi1* mutant alleles are not defective in bending response

Bending angle of rotated growing roots by 90 degrees was monitored after 2 and 24 hours. No bending defect was observed in *dmi1-1* and *dmi1-2* mutants in contrast to *cngc14-2* mutant allele. It was previously demonstrated that CNGC14 regulates root gravitropism in Arabidopsis, and thus *cngc14-2* is used here as a control. Whisker plots show 25% and 75% percentiles, median, minimum and maximum. Numbers under boxes denote sample size (n). ** $p < 0.01$, *** $p < 0.001$ (one-way ANOVA, Dunnett's multiple comparisons test vs. Col-0). The data represent two biological replicates.

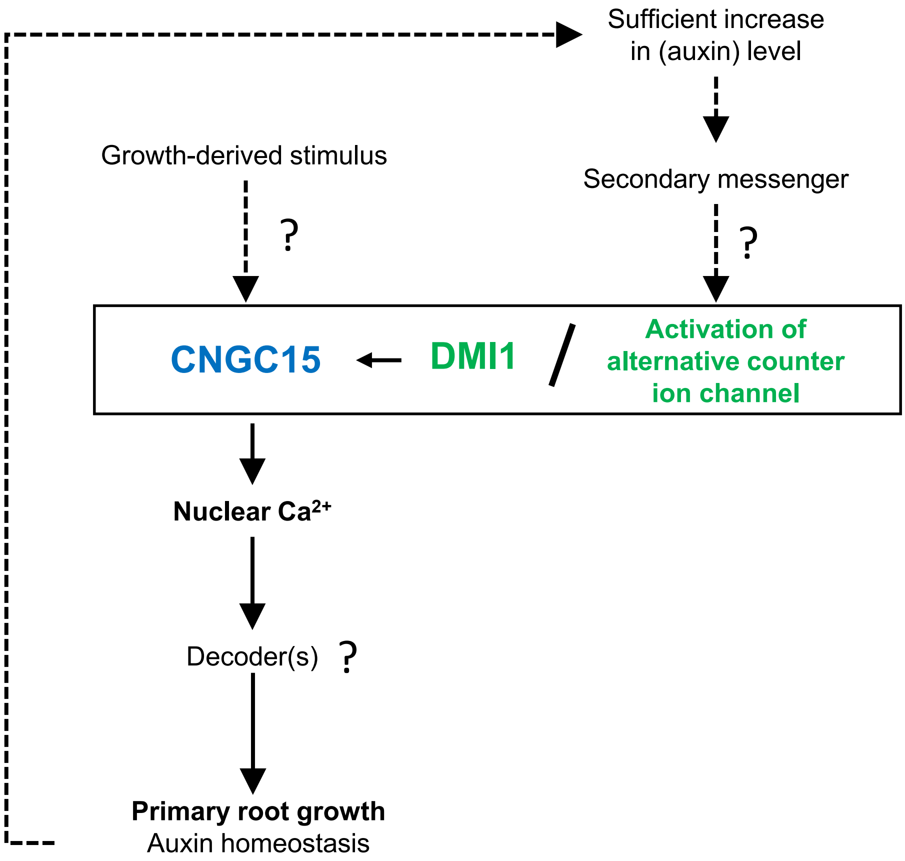

Supplementary Figure 17 | Nuclear calcium signals modulated by DMI1 are correlated with root development.

An endogenous growth-derived stimulus induces nuclear Ca^{2+} signals through DMI1 and CNGC15. The signal is interpreted by an unidentified Ca^{2+}-sensing protein, which translates the information to downstream effectors. This signal is necessary to establish an adequate auxin homeostasis in the root apical meristem, and consequently achieve normal root development. Increasing auxin levels can bypass the absence of DMI1 to potentially activate an alternative counter-ion channel, and sustain physiological Ca^{2+} release.

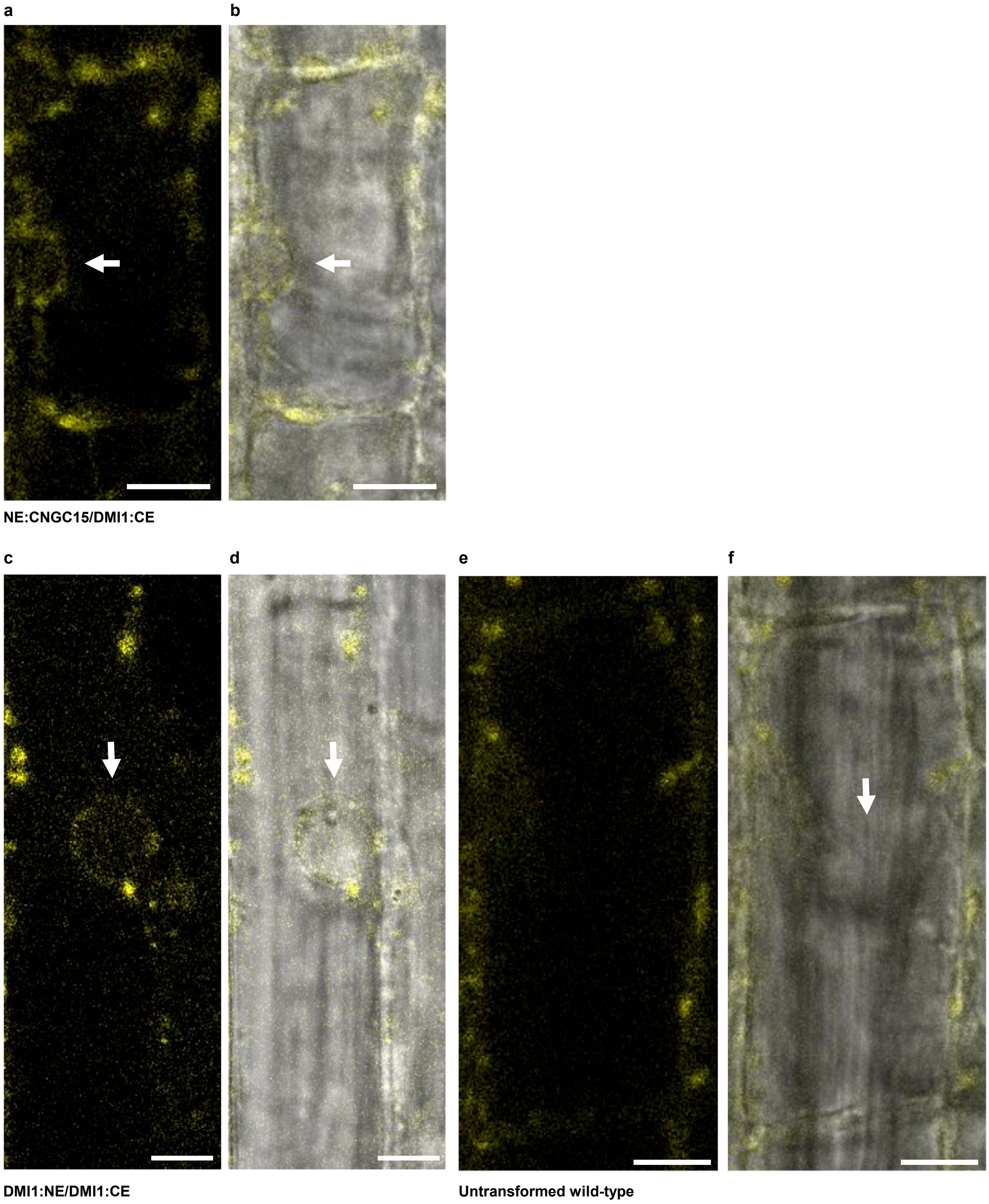

Supplementary Figure 18 | Bimolecular fluorescence complementation of CNGC15 with DMI1 in *A. thaliana* roots.

Laser scanning confocal microscopy images of 6 day-old *A. thaliana* root epidermal cells expressing N-terminal Venus (NE) fused to N-terminus of CNGC15, and C-terminal Venus (CE) fused to the C-terminus of DMI1 (**a**, **b**), DMI1:NE and DMI1:CE (**c**, **d**), background fluorescence in untransformed roots (**e**, **f**). Venus fluorescence detection on the right panels (**a**, **c**, **e**), and overlay picture with the bright field on the left panels (**b**, **d**, **f**). White arrows point to the nucleus. Scale bars represent 10 µm.

Figure	FUNCTION	Sequence ann	Name	Sequence (5'-3')		Efficiency (E)
S5	Genotyping	T-DNA	LbB1.3	ATTTTGCCGATTTCGGAAC	LB	
S5	Genotyping	T-DNA (SAIL)	LB3	TAGCATCTGAATTTCATAACCAATCTCG	LB	
S5	Genotyping	*dmi1-1*	7	CAGAGTTTGCATGGAACAATG	LP	
S5	Genotyping	*dmi1-1*	8	TGTGGTTGTGTTAGCAGAACG	RP	
S5	Genotyping	*dmi1-2*	9	TCATATGCAATCTCGAGCATC	LP	
S5	Genotyping	*dmi1-2*	10	GAGTTATCCTCTGACTCGGGG	RP	
S5	Genotyping	*cngc14-2*		TAAGAATCCAAGTGGCCACAC	LP	
S5	Genotyping	*cngc14-2*		TGTTTCACGTAAAGTCAAACCC	RP	
S2, S6	RT-qPCR	*CNGC15*	CNGC15f	CAAGACTGAGCTGAGGGCAA	fwd	100.1% (T_a = 58 ºC)
S2, S6	RT-qPCR	*CNGC15*	CNGC15re	CCACCATTCACTGCTAGCCT	rev	
S2, S5	RT-qPCR	*DMI1*	DMI1fwd	GTTATCGCCTTGCGGGAATG	fwd	111.1% (Ta = 58 ºC)
S2, S5	RT-qPCR	*DMI1*	DMI1rev	ACTGACTTGAGGCGATGACA	rev	
S2, S5, S12	RT-qPCR	*UBOX*	UBOXfwd	TGCGCTGCCAGATAATACACTATT	fwd	100.8% (Ta = 56 ºC)
S2, S5, S12	RT-qPCR	*UBOX*	UBOXrev	TGCTGCCCAACATCAGGTT	rev	105.0% (Ta = 58 ºC)
S12	RT-qPCR	*PIN1*	PIN1fwd	TACTCCGAGACCTTCCAACTACG	fwd	100.2% (Ta = 58 ºC)
S12	RT-qPCR	*PIN1*	PIN1rev	TCCACCGCCACCACTTCC	rev	
S12	RT-qPCR	*GH3-8*	GH3-8fwd	AGCCAAGTGTCCAATTCCTGA	fwd	98.0% (Ta = 58 ºC)
S12	RT-qPCR	*GH3-8*	GH3-8rev	CACATTGGACGTCAAGTCCTCTA	rev	
S12	RT-qPCR	*PURIN7*	PURIN7fw	TTTTGCCTCTGTGGCTGCTA	fwd	103% (Ta = 58 ºC)
S12	RT-qPCR	*PURIN7*	PURIN7re	TTGACCTCACACACTGAGCC	rev	
S12	RT-qPCR	*PID*	PIDfwd	ACTAAAAGACTTGGGTCACGGCGAG	fwd	100% (Ta = 58 ºC)
S12	RT-qPCR	*PID*	PIDrev	GTAATCGAACGCCGCTGGTTTGTTAC	rev	
S12	RT-qPCR	*PLT1*	PLT1fwd	TAGCGTCCAATCAAACGATG	fwd	97.1% (Ta = 58 ºC)
S12	RT-qPCR	*PLT1*	PLT1rev	CGGATGGTGAAGCTTTGTC	rev	
S12	RT-qPCR	*PLT2*	PLT2fwd	CAACGACAATATCGACAACCC	fwd	101.1% (Ta = 58 ºC)
S12	RT-qPCR	*PLT2*	PLT2rev	CGTTGGTTTGATGAATGTCG	rev	
S5	RT-PCR	*DMI1*	1	TGACTTGAGGCGATGACAACA	fwd	
S5	RT-PCR	*DMI1*	2	CGAGTCAATCCCCGAGTCAG	rev	
S5	RT-PCR	*DMI1*	3	GGTGAAAAGAAAAACCACCCCA	rev	
S5	RT-PCR	*DMI1*	4	CTCCCATATCTGCGCAAGACC	rev	

Supplementary Table 1: Primers used in this study

Figure	Construct			
	position 1	position 2	position 3	position 4
Supplementary Fig. 1	pNOS:BAR:tNOS	pAtUBI10:GFP:T35S	pAtUBI10:NESmcherry:T35S	
Supplementary Fig. 1 &11	pNOS:BAR:tNOS	pAtUBI10:gAtCNGC15:GFP:T35S	pAtUBI10:NESmcherry:T35S	
Supplementary Fig. 3	pNOS:BAR:tNOS	pAtCNGC15:GUS:T35S		
Supplementary Fig. 4	pNOS:BAR:tNOS	pAtDMI1:gAtDMI1:GFP:T35S		
Supplementary Fig. 4	pNOS:BAR:tNOS	pAtUBI10:AtDMI1:GFP:T35S		
Supplementary Fig. 4 & 8	pNOS:BAR:tNOS	pAtUBI10:GFP:T35S		
Supplementary Fig. 11	pNOS:BAR:tNOS	pAtUBI10:gAtDMI1:T35S		
Supplementary Fig. 5	pNOS:BAR:tNOS	pLjUBI1: YFP-Nterm:AtCNGC15:T35S	pAtUBI10:AtDMI1:YFP-Cterm:TOCs	p35S:DsRed:t35S
Supplementary Fig. 5	pNOS:BAR:tNOS	pLjUBI1:DMI1:YFP-Nterm:T35S	pAtUBI10:AtDMI1:YFP-Cterm:TOCs	p35S:DsRed:t35S
Supplementary Fig. 5	pNOS:BAR:tNOS	pLjUBI1: YFP-Nterm:AtCNGC15-1:T35S	pAtUBI10:AtDMI1:YFP-Cterm:TOCs	p35S:DsRed:t35S

Supplementary Table 2: Golden Gate construct generated in this study

Abbreviations: 35S: cauliflower mosaic virus 35S, Ocs: octopine synthase, NOS: nopaline synthase, AtUBI10: A. thaliana ubiquitin 10, LjUBI1: Lotus japonicus ubiquitin 1, NES: nuclear exclusion signal, DsRed: Discosoma sp. red fluorescent protein
