# Supplementary material for: Nuclear calcium signatures are associated with root development
Source: Nat Commun. 2019 Oct 25;10:4865. doi: 10.1038/s41467-019-12845-8 (PMC6814746; doi:10.1038/s41467-019-12845-8)
Supplement: Supplementary file 2 — Description of Additional Supplementary Files [file 41467_2019_12845_MOESM2_ESM.pdf]

Description of Additional Supplementary Files

Supplementary Movie 1: Visualization with the red channel of the nuclear calcium spikes in Arabidopsis root meristem expressing the dual sensor R-GECO1.2-NLS/GGECO1.2-NES.

Supplementary Movie 2: Simultaneous visualization of calcium release in the nucleus and cytoplasm in Arabidopsis root meristematic cell expressing the dual sensor RGECO1.2-NLS/G-GECO1.2-NES.

Supplementary Data 1: Script developed to analyse the fall and rise times of the calcium spikes and calcium data.
