# Supplementary material for: Nuclear calcium signatures are associated with root development
Source: Nat Commun. 2019 Oct 25;10:4865. doi: 10.1038/s41467-019-12845-8 (PMC6814746; doi:10.1038/s41467-019-12845-8)
Supplement: Supplementary file 6 — Reporting Summary [file 41467_2019_12845_MOESM6_ESM.pdf]

nature research

Corresponding author(s): DBPR

Last updated by author(s): Sep 25, 2019

Reporting Summary

Nature Research wishes to improve the reproducibility of the work that we publish. This form provides structure for consistency and transparency in reporting. For further information on Nature Research policies, see Authors & Referees and the Editorial Policy Checklist .

Statistics

For all statistical analyses, confirm that the following items are present in the figure legend, table legend, main text, or Methods section.

n/a	Confirmed	
☐	☒	The exact sample size (*n*) for each experimental group/condition, given as a discrete number and unit of measurement
☐	☒	A statement on whether measurements were taken from distinct samples or whether the same sample was measured repeatedly
☐	☒	The statistical test(s) used AND whether they are one- or two-sided<br>*Only common tests should be described solely by name; describe more complex techniques in the Methods section.*
☒	☐	A description of all covariates tested
☐	☒	A description of any assumptions or corrections, such as tests of normality and adjustment for multiple comparisons
☐	☒	A full description of the statistical parameters including central tendency (e.g. means) or other basic estimates (e.g. regression coefficient) AND variation (e.g. standard deviation) or associated estimates of uncertainty (e.g. confidence intervals)
☐	☒	For null hypothesis testing, the test statistic (e.g. *F*, *t*, *r*) with confidence intervals, effect sizes, degrees of freedom and *P* value noted<br>*Give P values as exact values whenever suitable.*
☒	☐	For Bayesian analysis, information on the choice of priors and Markov chain Monte Carlo settings
☒	☐	For hierarchical and complex designs, identification of the appropriate level for tests and full reporting of outcomes
☒	☐	Estimates of effect sizes (e.g. Cohen's *d*, Pearson's *r*), indicating how they were calculated

Our web collection on statistics for biologists contains articles on many of the points above.

Software and code

Policy information about availability of computer code

Data collection	The commercially available ImageJ 1.48v was used to collect the data provided for Fig.1,2,3,4 and Supplementary fig. 6, 7, 8, 10, 11, 13, 14, 15, 16
Data analysis	GraphPad Prism (v5.01) was used to analyse the data in Fig. 1b,c, e-j; 2c,d,e,g; 3a,b,d; 4b,c,d as well as supplementary fig. 1, 2, 5c, 6a,6b,6d,6e,6f, 7, 8b, 10d,10h, 11b,11c, 12, 13, 14, 15, 16.<br>Custom code for calculating the fall and rise times of the calcium spiking was used. This code was developed in Python v2.7 and is available as a .zip archive upon request and will be publicly available from the on-line git repository gitlab upon manuscript acceptance. Code is OS independent but does require the use of a third party package (xlrd, available at https://pypi.org/project/xlrd/). A readme file showing example usage is supplied with the analysis scripts. The script is made available under an MIT license.

For manuscripts utilizing custom algorithms or software that are central to the research but not yet described in published literature, software must be made available to editors/reviewers. We strongly encourage code deposition in a community repository (e.g. GitHub). See the Nature Research guidelines for submitting code & software for further information.

Data

Policy information about availability of data

All manuscripts must include a data availability statement. This statement should provide the following information, where applicable:

- Accession codes, unique identifiers, or web links for publicly available datasets
- A list of figures that have associated raw data
- A description of any restrictions on data availability

Source data are provided for Fig. 1, 2, 3, 4 and Supplementary Figs. 1, 2, 5c, 6, 7, 8b, 9, 10d, 10h, 11b, 11c, 12, 13, 14, 15, 16. All other data are available from the corresponding author and material upon reasonable request.

Field-specific reporting

Please select the one below that is the best fit for your research. If you are not sure, read the appropriate sections before making your selection.

[x] Life sciences [ ] Behavioural & social sciences [ ] Ecological, evolutionary & environmental sciences

For a reference copy of the document with all sections, see nature.com/documents/nr-reporting-summary-flat.pdf

Life sciences study design

All studies must disclose on these points even when the disclosure is negative.

Sample size	Sample sizes for each experiment was chosen as large as possible and in accordance with previous established protocols in the field and experience.
Data exclusions	For calcium imaging recordings, the root which grew out of focus were excluded from the analysis.<br>For root phenotyping, the roots were excluded from the analyses if the seed did not germinate or the plate were contaminated.
Replication	All assays were independently repeated successfully and all attempts of replication by different people in the group were successful.
Randomization	Sampling for A. thaliana root phenotyping were grown as follow: 6 wild type, 6 mutants per plate. The plates were randomly positioned on the shelves. Samples for calcium imaging were randomly picked from plates. Sampling for nodulation and mycorrhization were randomly positioned on the shelves.
Blinding	Blinding was not relevant for the experiments in which the data were collected using image analysis software and code, not manually. The scoring of symbioses phenotype was performed in blind without the experimenter knowledge of lines.

Reporting for specific materials, systems and methods

We require information from authors about some types of materials, experimental systems and methods used in many studies. Here, indicate whether each material, system or method listed is relevant to your study. If you are not sure if a list item applies to your research, read the appropriate section before selecting a response.

Materials & experimental systems

n/a	Involved in the study	
[x]	[ ]	Antibodies
[x]	[ ]	Eukaryotic cell lines
[x]	[ ]	Palaeontology
[x]	[ ]	Animals and other organisms
[x]	[ ]	Human research participants
[x]	[ ]	Clinical data

Methods

n/a	Involved in the study	
[x]	[ ]	ChIP-seq
[x]	[ ]	Flow cytometry
[x]	[ ]	MRI-based neuroimaging
